# A safety-aware intake modeling framework for translating edible algal biomass into nutritional bioproducts

**DOI:** 10.1007/s44307-026-00129-4

**Published:** 2026-08-10

**Authors:** Yu Wang, Garrett Roell, Rica Dela Cruz, Michaela Durďáková, Jessica Maruwan, Kexin Rong, Rachel Novotny, Monica Esquivel, Lynne Wilkens, Wei Wen Su, Tao Yan, Kacie Ho, Zhi-Yan Du

**Affiliations:** 1 Department of Molecular Biosciences and Bioengineering, University of Hawaiʻi at Mānoa, Honolulu, Hawaiʻi 96822 USA; 2 Department of Human Nutrition, Food and Animal Sciences, University of Hawaiʻi at Mānoa, Honolulu, Hawaiʻi 96822 USA; 3 Department of Chemistry and Biochemistry, Mendel University in Brno, Zemedelska 1, 61300 Brno, Czech Republic; 4 Research Center for Applied Molecular Oncology, Masaryk Memorial Cancer Institute, Zluty Kopec 7, 65653 Brno, Czech Republic; 5 Water Resources Research Center, University of Hawaiʻi at Mānoa, Honolulu, Hawaiʻi 96822 USA; 6 University of Hawaiʻi Cancer Center, Honolulu, Hawaiʻi 96822 USA

**Keywords:** Edible algal biomass, Microalgae, Algal biotechnology, Omega-3 fatty acids, Safety-aware modeling, Nutritional bioproducts

## Abstract

Edible algal biomass has emerged as a promising platform for nutritional bioproduct development, but its practical translation depends on compositional variability, intake feasibility, and safety constraints. Here, we integrated biochemical profiling, trace element screening, age-specific intake modeling, and community consumption context to evaluate commercially available and supplier-provided microalgal and seaweed products within realistic application boundaries. Selected products differed substantially in macronutrient composition, fatty acid profiles, pigments, polyphenols, and mineral composition, indicating product- and species-specific roles as nutritional biomass rather than interchangeable ingredients. Across species and age groups, approximately 0.7 to 3.1 g dry biomass provided 20% of age-specific alpha-linolenic acid intake, with approximately 1 g sufficient for several products. Multi-species allocations improved omega-3 representation while remaining within practical intake limits and trace element-based screening thresholds. Community consumption data from Pacific Island children showed low and episodic seaweed intake, highlighting a gap between biomass potential and routine dietary use. These findings support safety-aware modeling as a preliminary screening and decision-support strategy for evaluating edible algal biomass before further product-specific validation.

**Supplementary Information:**

The online version contains supplementary material available at 10.1007/s44307-026-00129-4.

## Introduction

Ensuring access to nutritionally adequate diets under increasing environmental and supply chain pressures is a defining challenge for contemporary food systems (Crippa et al. 2021). Although global food production has expanded substantially, nutrient inadequacy remains widespread across age groups, particularly for nutrients such as omega-3 fatty acids and other micronutrients that are unevenly distributed across commonly consumed foods (FAO 2023; National Institutes of Health, Office of Dietary Supplements 2025). At the same time, climate change, land limitation, freshwater scarcity, and pressure on marine resources are constraining the long-term stability of conventional agriculture and fisheries (FAO 2023). Together, these trends highlight the need not only for more food, but for foods that can deliver high nutritional value with relatively low resource demands (Kumar et al. 2022; Diaz et al. 2023).

**Fig. 1:**
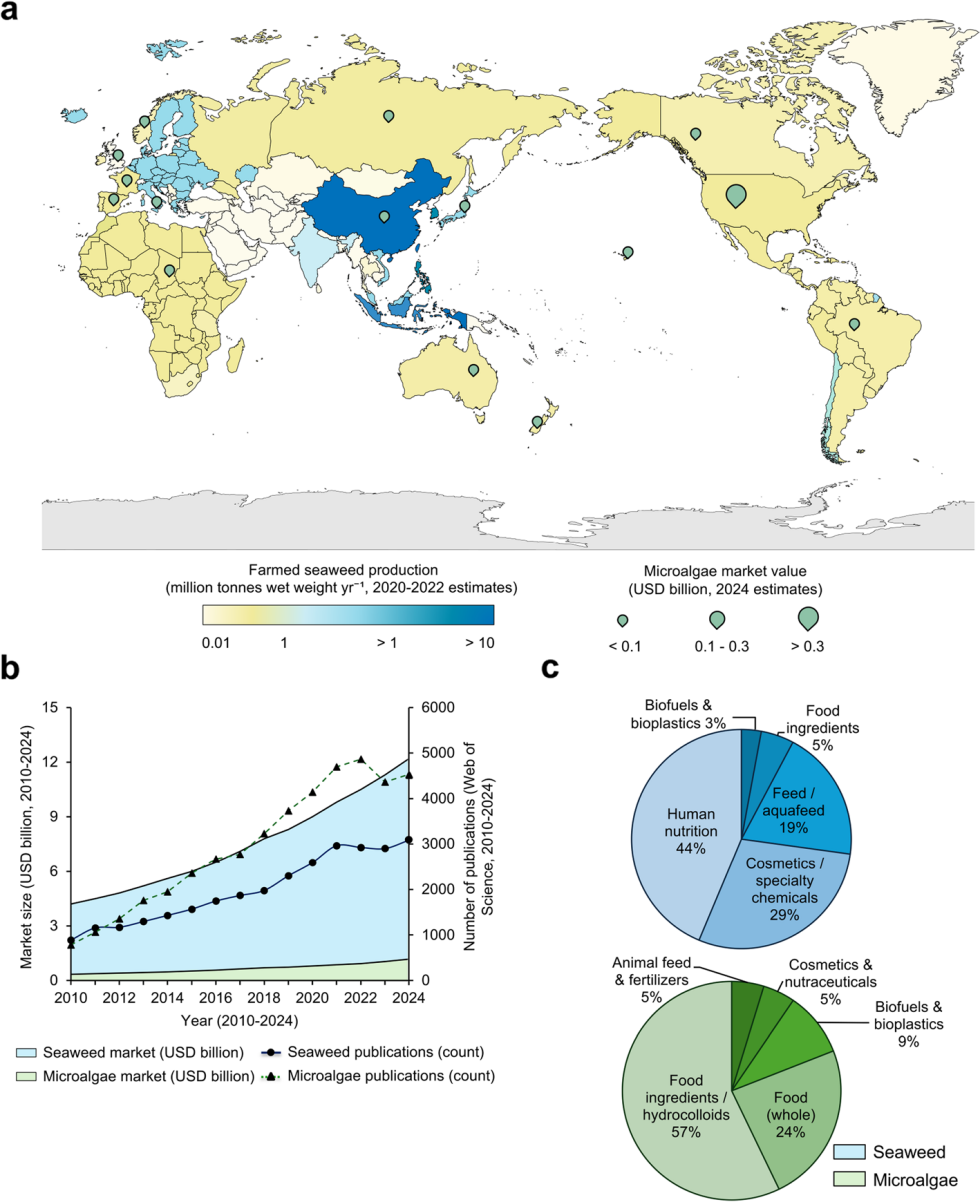
Global production, market expansion, and utilization pathways of edible algae. **a**, Farmed seaweed production by country (mean 2020–2024; million tonnes wet weight) and estimated microalgae market value (2024; USD billions). Countries without reported production are shown in light yellow; Antarctica is shown in grey. **b,** Global market growth of seaweed and microalgae (2010–2024; USD billions, left axis) and annual publication output indexed in Web of Science (right axis), illustrating parallel expansion in commercial activity and scientific research. **c,** Estimated allocation of seaweed and microalgal biomass across major application sectors in 2024, synthesized from recent market assessments and review literature

In response, increasing attention has turned to edible algal biomass as a platform for nutritional bioproduct development. Edible algae, including both microalgae and macroalgae, have emerged as promising candidates because they can be cultivated in marine or controlled systems without dependence on arable land and can accumulate protein, omega-3 fatty acids, minerals, and bioactive compounds (Kumar et al. 2022; Diaz et al. 2023). This direction is consistent with broader biotechnology efforts to convert renewable biological feedstocks into single-cell protein and other nutritional bioproducts (Zhuang et al. 2024). In this study, the term “nutritional bioproduct” refers to edible algal biomass or biomass-derived ingredients intended to contribute nutrients, bioactive compounds, or functional value within food systems. Some algal-derived ingredients are already used commercially, particularly as sources of long-chain omega-3 fatty acids and pigments, demonstrating their nutritional and industrial relevance (Adarme-Vega et al. 2014; Torres-Tiji et al. 2020; Gao et al. 2024). Ongoing advances in algal biotechnology, synthetic biology, and molecular biology continue to expand the toolkit available for future algal bioproduct development (Do et al. 2025; Guo et al. 2026). However, the value of edible algae cannot be inferred from biomass productivity or nutrient density alone. Their contribution to human nutrition depends on realistic intake levels, safety constraints, product form, and incorporation into foods that people routinely consume (Fig. 1). This pattern creates a need to evaluate algae not only as nutrient-rich biomass, but as edible biomass resources with defined nutritional, safety, and application boundaries (Casas-Agustench et al. 2024; Van der Stricht et al. 2024).

A central reason is that nutrient density alone does not determine dietary relevance. Foods are consumed within cultural, behavioral, economic, and regulatory contexts, and their contribution to nutrition depends not only on biochemical composition, but also on realistic intake levels, safety constraints, and compatibility with existing dietary patterns (Meixner et al. 2023; Van der Stricht et al. 2024; Varela et al. 2024). Many studies on algae emphasize nutrient composition or production efficiency. Still, fewer evaluate how edible algae function within dietary frameworks that consider age-specific nutrient needs, contaminant exposure, and observed consumption patterns (Herrero et al. 2021; Gao et al. 2024; Casas-Agustench et al. 2024). As a result, a substantial gap remains between the documented nutritional potential of algae and their practical use as safe, food-compatible nutritional biomass sources.

This gap is especially important in island and coastal food systems, where import dependence, limited agricultural land, and vulnerability to supply disruptions increase the value of locally producible, nutrient-dense foods (Diaz et al. 2023; FAO 2023). Hawaiʻi provides a useful case study in this context. Edible algae are locally cultivable and nutritionally promising, and seaweed has cultural relevance in some Pacific communities, yet its broader dietary contribution remains limited. At the same time, global patterns in algal production and commercialization reveal a wider disconnect between nutritional promise and food-based use, with seaweed production concentrated in a few regions and microalgae often marketed more as supplements or sources of isolated compounds than as ingredients integrated into everyday diets (Torres-Tiji et al. 2020; Araújo et al. 2021; Liu et al. 2022; FAO 2022, 2023, 2024). In parallel, microalgae are increasingly explored for non-human nutritional applications, including feed additives for livestock sustainability, further illustrating the broad but fragmented utilization landscape of algal biomass (Zhu et al. 2024).

Using species relevant to Hawaiʻi as a case study, we combine compositional analysis, conservative safety screening, age-specific dietary modeling, and community consumption data to assess how algae can contribute to nutrient intake at food-compatible inclusion levels. We quantify macronutrients, fatty acids, bioactive compounds, and trace elements, model gram-scale intake requirements across life stages using established dietary reference values (National Institutes of Health, Office of Dietary Supplements 2025; U.S. Department of Agriculture & U.S. Department of Health And Human Services 2020), and contextualize these findings within observed consumption patterns and food-based applications. Rather than positioning algae as supplements, replacement foods, or fully validated products, this study evaluates selected edible algal products as biomass sources that can be screened within safety-aware intake limits. By integrating nutrient density, trace element-based screening, intake feasibility, and dietary context, this framework provides a preliminary decision-support approach for identifying promising algal biomass candidates that require further product-specific validation before nutritional bioproduct development.

## Materials and methods

This study evaluated edible algal biomass as a platform for nutritional bioproduct development by integrating compositional analysis, conservative safety screening, age-specific intake modeling, community consumption data, and food-compatible incorporation trials. The objective was to determine whether algal biomass can contribute nutritionally meaningful amounts of key nutrients at realistic intake levels while remaining within safety-aware application boundaries. All compositional analyses were performed on dried biomass and expressed on a dry-weight (DW) basis (µg g^−1^ DW or % DW). Unless otherwise stated, measurements were conducted using three independently weighed analytical subsamples from each dried biomass product, with technical triplicate measurements (n = 3) for each subsample. These replicates capture within-product analytical variability but do not represent independent production batches.

### Global production and utilization context for edible algal biomass

Country-level seaweed production data used in Fig. 1 were obtained from the Food and Agriculture Organization of the United Nations (FAO) fisheries and aquaculture database (FAO 2022, 2024). Macroalgae production was calculated as the mean annual output for 2020–2022 and reported as million tonnes wet weight (Supplementary Table S1). Countries with no reported production were classified accordingly for visualization. Country-level algal market values for 2024 were compiled from industry reports (Grand View Research 2022; Introspective Market Research 2024; Spherical Insights 2025). Where multiple estimates were available, values were harmonized to a common reporting year and scope. Global market size time series for seaweed and microalgae were compiled from industry reports, and publication output was quantified using Web of Science searches for “seaweed OR macroalgae” and “microalgae OR microalgal.” Downstream biomass allocation across application sectors was synthesized from market assessments and review of the literature. Categories were harmonized as human nutrition, food ingredients or hydrocolloids, feed or aquafeed, cosmetics or specialty chemicals, and biofuels or bioplastics. Reported proportions represent synthesized estimates rather than direct measurements. Global patent data (Supplementary Fig. S1) were retrieved from Lens.org (https://www.lens.org, accessed 18 November 2025) using the same keyword queries, restricted to 2010–2024 and including all jurisdictions and document types.

### Biochemical diversity of edible algal biomass

Edible microalgae and seaweed species were selected to represent taxa commonly used or proposed for human consumption. Microalgae included *Arthrospira platensis*, *Chlorella* sp., *Dunaliella salina* (product A), *Dunaliella salina* (product B), *Haematococcus pluvialis*, *Nannochloropsis gaditana*, *Nannochloropsis salina*, *Porphyridium purpureum*, *Schizochytrium limacinum*, and *Tetraselmis chuii*. One red seaweed species, *Gigartina skottsbergii*, was included for comparison. Biomass was obtained from commercially available products and supplier-provided research samples representing species commonly used or proposed for human consumption (Supplementary Table S2). The analyzed materials were treated as product-level examples of edible algal biomass rather than definitive species-level reference materials. Because algal composition can vary with strain, cultivation conditions, harvest stage, drying method, processing, storage, and supplier, the results should be interpreted as an exploratory comparison of selected biomass products and not as comprehensive species-level nutritional profiles. To avoid brand-specific inference and ensure strain-neutral reporting, samples from the same species obtained from different production systems were labeled using neutral identifiers, for example, *Dunaliella salina* (A) and (B). *Schizochytrium limacinum* was included because it is widely used as a commercial source of DHA-rich biomass for nutritional applications, although it is taxonomically classified as a thraustochytrid rather than a photosynthetic microalga. Biomass was harvested at late growth or commercial maturity, dried to constant weight, and milled to a fine powder prior to analysis.

### Macronutrient analysis

Protein, lipid, and carbohydrate contents were quantified for each algal sample using standard analytical methods. Protein content was determined using a modified Lowry assay following protein precipitation with trichloroacetic acid and reaction with Folin-Ciocalteu reagent, with absorbance measured at 750 nm using bovine serum albumin standards for calibration (Slocombe et al. 2013). Total lipid content was quantified gravimetrically following chloroform–methanol extraction (Du et al. 2018). Carbohydrate content was quantified using the phenol–sulfuric acid method and expressed as glucose equivalents (Dubois et al. 1956). Macronutrient contents were expressed as a percentage of dry weight.

Total lipids were converted to fatty acid methyl esters (FAMEs) using acid-catalyzed transesterification and analyzed by gas chromatography with flame ionization detection (Agilent 8860) using a polar capillary column suitable for marine fatty acid separation. Individual fatty acids were identified using a 37-component FAME standard mix (Sigma) and quantified as percentages of the total detected fatty acids. Fatty acids were grouped into saturated fatty acids (SFA), monounsaturated fatty acids (MUFA), omega-3 polyunsaturated fatty acids (omega-3 PUFA), and omega-6 PUFA.

Calibration was performed using assay-specific external standards as described above. For compositional assays, calibration curves were generated using bovine serum albumin for protein, glucose for carbohydrate, and the 37-component FAME standard mix for fatty acid identification. Elemental analysis used instrument calibration and laboratory blanks to define detection limits. Recovery experiments, certified reference materials, assay-specific limits of quantification, and independent production-batch validation were not available for all assays; therefore, the compositional results are interpreted as comparative screening data rather than official nutrient-label values.

### Pigment and bioactive compound analysis

Photosynthetic pigments and phenolic compounds were quantified to characterize bioactive composition. Chlorophyll a and b were extracted using acetone:DMSO solvent and quantified spectrophotometrically (Porra 2002). Carotenoids were extracted and analyzed using high-performance liquid chromatography with detection at 455 nm, and β-carotene and astaxanthin were quantified using external standards (Lourenço-Lopes et al. 2022). Total polyphenols were quantified using the Folin-Ciocalteu method and expressed as gallic acid equivalents (Kolackova et al. 2020). Relative contributions of pigments and polyphenols were calculated as a fraction of total measured bioactive compounds.

### Heavy metal and essential mineral composition and associated safety screening metrics

Elemental composition was determined following acid digestion of dried biomass and quantified using inductively coupled plasma-based analysis. Analysis was conducted at the University of Hawaiʻi at Mānoa, Water Resources Research Center, using the Thermo Scientific iCAP 6300 DUO ICP-OES. Nutritionally relevant minerals and trace elements of food-safety relevance, including lead (Pb), total arsenic (As), and total mercury (Hg), were measured. Instrument detection limits were defined using laboratory blanks, and concentrations below detection were classified as non-detects.

To contextualize elemental concentrations in relation to dietary exposure, conservative maximum intake screening thresholds were estimated. Upper-bound elemental concentrations were defined as mean plus two standard deviations for detected elements, or as the detection limit for non-detects. Maximum screening intake (g DW day⁻^1^) was estimated by dividing applicable health-based intake benchmarks by the corresponding upper-bound elemental concentrations. Screening benchmarks included the US Food and Drug Administration interim reference level for lead, the US Environmental Protection Agency reference dose for inorganic arsenic (Agency for Toxic Substances And Disease Registry 2007; Flannery & Middleton 2022; U.S. Environmental Protection Agency IRIS Program 2025) (applied conservatively to total arsenic), and the European Food Safety Authority tolerable intake for mercury (EFSA Panel on Contaminants in the Food Chain (CONTAM), 2012).

A standardized child reference body weight of 20 kg was used to convert body-weight-based exposure thresholds into practical intake limits. This value was derived from CDC weight-for-age growth-chart reference data and approximates the median body weight of a 6-year-old child (U.S. Centers for Disease Control And Prevention 2024). Because the framework was designed for comparative screening and decision support rather than individualized toxicological risk assessment, a single reference body weight was used for these calculations.

Because arsenic was measured as total arsenic, the inorganic arsenic benchmark was applied as a conservative upper-bound screening assumption. This approach was intended as a precautionary screening assumption and should not be interpreted as a refined arsenic risk assessment. The analysis did not include arsenic speciation, iodine quantification, cadmium quantification, or product-level contaminant validation across independent batches.

### Safety-aware intake modeling of omega-3 contribution from edible algal biomass

Age-specific nutrient modeling was conducted to evaluate the potential contribution of edible algal biomass to omega-3 fatty acid intake across life stages. Modeling was based on dietary reference intakes for alpha-linolenic acid (ALA), which is the omega-3 fatty acid with an established adequate intake value in the US dietary reference framework (National Institutes of Health, Office of Dietary Supplements 2025; U.S. Department of Agriculture & U.S. Department of Health And Human Services 2020, 2026).

ALA was used as the primary reference benchmark because it has age- and sex-specific adequate intake values, allowing comparison across life stages. Eicosapentaenoic acid (EPA) and docosahexaenoic acid (DHA) were retained as long-chain omega-3 compositional attributes because several algal products are nutritionally valuable sources of these fatty acids; however, they were not used as formal DRI-based adequacy targets because formal US dietary reference intake values are not established for EPA or DHA (Troesch et al. 2020; Liu et al. 2022; National Institutes of Health, Office of Dietary Supplements 2025).

For Fig. 3, the dry biomass intake required to provide 20% of the age-specific ALA reference intake was calculated for each algal product. This 20% value was used as a standardized visualization benchmark. For clarity of presentation, panels a-d highlight representative high-performing species requiring the lowest biomass inputs to achieve the benchmark, illustrating whether selected algal biomass products could provide a meaningful partial omega-3 contribution at low inclusion levels. It was not used as the primary optimization target in the final allocation model and should not be interpreted as a dietary recommendation. In addition, the model was based on compositional values measured in dried biomass and did not account for nutrient retention during food processing or differences in nutrient bioavailability following consumption.

### Constraint-based dietary modeling framework

The model first identified allocations satisfying the minimum ALA contribution target, total biomass constraint, user-defined dietary restrictions, and trace element-based screening constraints. When feasible within the remaining biomass allowance (≤ 2.0 g day⁻^1^), allocations were further supplemented with EPA- and DHA-containing products to achieve the practical representation targets described above. When multiple feasible allocations satisfied the core nutritional and screening constraints, product selection was further informed by user-defined nutrient priorities and contextual requirements.

After these core constraints were satisfied, feasible allocations were further prioritized based on total omega-3 contribution and user-defined nutrient priorities within the practical biomass cap. Condition-specific factors such as iodine sensitivity, pregnancy or lactation status, medication interactions, and purine-related concerns were handled through the advisory layer rather than as universal optimization constraints because applicable thresholds may vary by individual health status, product composition, and clinical context. Additional details regarding advisory-layer implementation are provided in the Supplementary Methods section S9.

The decision variable ${x}_{i}$ was defined as the dry biomass intake of algal product i in g day^−1^. Nutrient and trace element delivery were calculated as $\sum_{i}{x}_{i}{C}_{ij}$, where ${C}_{ij}$ represents the concentration of nutrient or trace element j in product i on a dry-weight basis. The model first identified allocations satisfying the minimum ALA, EPA, DHA, total biomass, user-defined dietary restrictions, and trace element-based screening constraints. When multiple feasible allocations satisfied the core nutritional and screening constraints, product selection was further informed by user-defined nutrient priorities and contextual requirements. Full parameter definitions, units, constraints, and representative output tables are provided in the Supplementary Methods section S8 and Supplementary Tables S4 and S7.

Age, sex, and body weight (Fryar et al. 2021; U.S. Centers for Disease Control And Prevention 2024) were incorporated to ensure that modeled intake levels remained within realistic food consumption ranges across life stages. Additional details on the model structure, mathematical formulation, workflow, sensitivity analysis, and example outputs are provided in Supplementary Methods section S8-S9, Supplementary Table S7, and Supplementary Figs. S3 and S4. An interactive web-based implementation of the modeling framework was developed to generate personalized intake scenarios based on user inputs and is available online (see Tool availability).

The dietary modeling framework included voluntary pilot submissions in which participants provided age, sex, body weight, and dietary constraints for the purpose of generating personalized nutrition reports. No identifiable personal information was collected. The study protocol was reviewed by the University of Hawaiʻi Institutional Review Board and was determined to be exempt from human subjects research oversight (IRB Protocol ID: 2026–00185).

### Application context for edible algae in Pacific Island communities

Community-level algae consumption patterns were evaluated using dietary record data from the Children’s Healthy Living (CHL) Program collected across Pacific Island jurisdictions in 2013, 2015, and 2019. The analysis included children aged 2–8 years with available diet records across Palau, Yap, Guam, the Commonwealth of the Northern Mariana Islands, Chuuk, Pohnpei, Kosrae, the Republic of the Marshall Islands, American Samoa, Hawaiʻi, and Alaska. The full CHL jurisdiction-level dataset included 6,055 children with diet records, and the Hawaiʻi subset included 881 children across the analyzed Hawaiʻi communities.

Seaweed intake was defined as any reported consumption of seaweed or seaweed-containing food items recorded in the diet records, including seaweed, nori, kombu, hijiki, wakame, furikake, musubi, sushi, miso soup, and related mixed foods. Because the purpose of this analysis was to evaluate exposure and dietary familiarity rather than nutrient intake from seaweed, children were classified as consumers or non-consumers based on whether any seaweed-containing item was reported. Portion-size data were not used to estimate nutrient contribution because seaweed intake was low, episodic, and often recorded as part of mixed foods. Therefore, the analysis focused on the prevalence of reported seaweed consumption rather than quantitative seaweed intake.

Only de-identified, aggregated data were analyzed. The proportion of children reporting seaweed intake was summarized by jurisdiction and, for Hawaiʻi, by community. Differences in prevalence across regions were evaluated using chi-square tests of independence followed by post-hoc pairwise proportion tests with Holm correction.

### Food-compatible translation of algal biomass into nutritional applications

To evaluate whether modeled intake levels were compatible with real food formats, algal biomass was incorporated into commonly consumed foods at low inclusion levels. Prototype products included algal-enriched noodles and desserts prepared using *Arthrospira platensis* and *Chlorella* sp. at inclusion levels designed to deliver gram-scale algae intake per serving. Inclusion levels ranged from approximately 1–2% dry weight substitution, corresponding to approximately 1 g dry algal biomass per serving. Product preparation was conducted in a certified kitchen following local food safety standards. These trials were conducted to assess the feasibility of delivering nutritionally relevant algae intake through commonly consumed food formats rather than through bulk algae consumption.

## Results

### Global production and utilization context for edible algal biomass

To position edible algae within a broader food-system context, we first examined global production, market expansion, and downstream utilization patterns (Fig. 1; Supplementary Fig. S1; Supplementary Table S1). Farmed seaweed production remained concentrated in a limited number of countries, whereas the commercial value of microalgae was distributed primarily through higher-value product markets rather than bulk food use (Fig. 1a; Supplementary Table S1). Market growth and publication activity increased in parallel over the past decade, indicating expanding scientific and commercial attention to algae (Fig. 1b; Supplementary Fig. S1). However, estimated downstream allocation showed that only a minority of algal biomass was directed toward direct human nutrition, with substantial fractions allocated to hydrocolloids, feed, and other industrial applications (Fig. 1c; Supplementary Table S1). These patterns indicate that although algal production and commercialization are expanding, food-based utilization remains limited.

### Biochemical diversity of edible microalgal and seaweed biomass

**Fig. 2:**
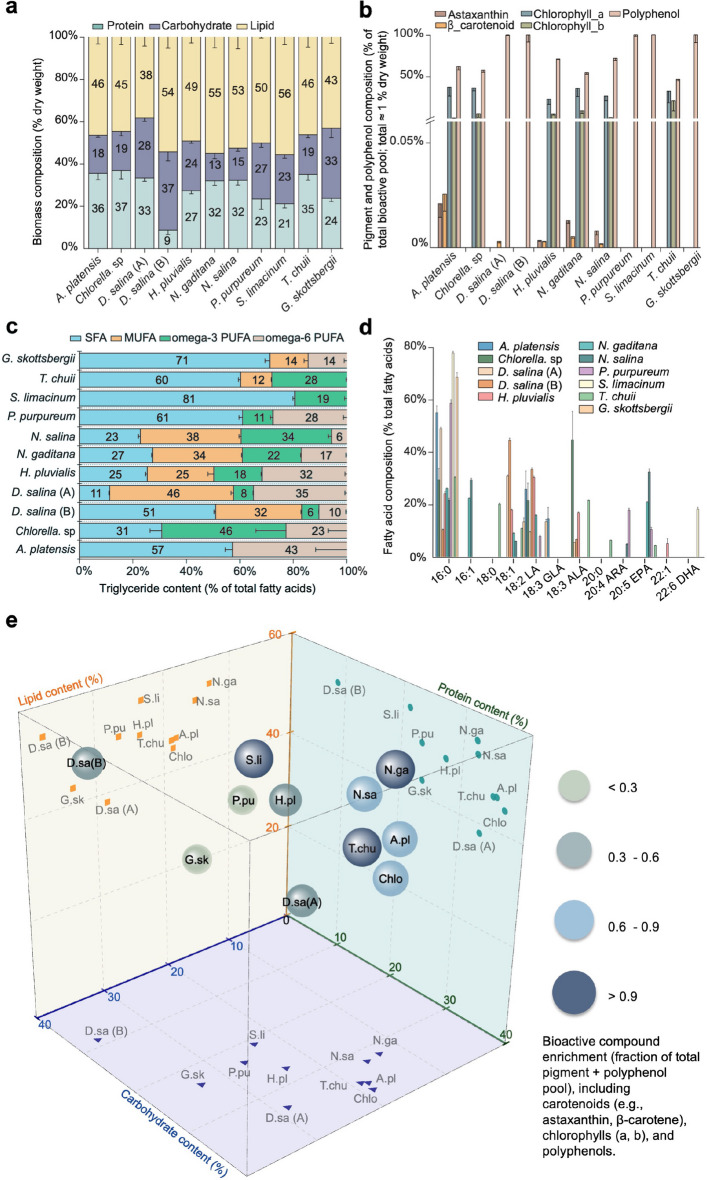
Nutritional composition and bioactive diversity across representative microalgae and seaweed species. **a,** Macronutrient composition (protein, carbohydrate, and lipid; % dry weight) across eleven commonly consumed microalgae and seaweed species. **b,** Relative distribution of measured bioactive compounds (% of total quantified bioactive fraction), including astaxanthin, β-carotene, chlorophylls a and b, and total polyphenols; the quantified bioactive pool represents ~ 1% of dry biomass. **c,** Fatty acid class distribution (% total fatty acids), including saturated (SFA), monounsaturated (MUFA), omega-3 polyunsaturated (omega-3 PUFA), and omega-6 PUFA fractions. **d,** Detailed fatty acid composition (% total fatty acids), highlighting nutritionally relevant species-specific variation in major fatty acids. **e,** Integrated three-dimensional representation of macronutrient composition (protein, carbohydrate, lipid), with bubble shading indicating relative enrichment of bioactive compounds, enabling cross-species comparison of nutritional and functional attributes. Species abbreviations: *A. platensis*/A.pl (*Arthrospira platensis*); Chlo (*Chlorella* sp.); *D. salina* (A, B)/D.sa (A, B) (*Dunaliella salina* variants); *H. pluvialis*/H.pl (*Haematococcus pluvialis*); *N. gaditana*/N.ga (*Nannochloropsis gaditana*); *N. salina*/N.sa (*Nannochloropsis salina*); *P. purpureum*/P.pu (*Porphyridium purpureum*); *S. limacinum*/S.li (*Schizochytrium limacinum*); *T. chuii*/T.chu (*Tetraselmis chuii*); *G. skottsbergii*/G.sk (*Gigartina skottsbergii*)

We next quantified the nutritional composition of the algal products included in this study, which comprised commercially available and supplier-provided microalgal and seaweed biomasses representing species used or proposed for human consumption (Supplementary Table S2; Fig. 2; Supplementary Fig. S2). Macronutrient composition varied substantially across taxa (Fig. 2a). Protein content ranged from less than 20% to more than 35% dry weight (DW), with *Arthrospira platensis* and *Chlorella* sp. showing the highest values at 35.54 ± 3.00% DW and 36.88 ± 3.97% DW, respectively. Carbohydrates predominated in *Gigartina skottsbergii* and *Dunaliella salina* (B), accounting for approximately 33–37% DW. Lipid content showed the greatest interspecific variability, ranging from 38 to 56% DW, with *Schizochytrium limacinum* exhibiting the highest lipid fraction (55.61 ± 3.51% DW).

Bioactive compounds, including pigments and polyphenols, collectively accounted for approximately 1% of dry biomass but differed markedly in composition among species (Fig. 2b; Supplementary Fig. S2). *Nannochloropsis gaditana* and *Tetraselmis chuii* showed the highest combined chlorophyll and polyphenol fractions, whereas carotenoids were present at comparatively lower levels, with β-carotene detected in *Arthrospira platensis* and trace astaxanthin in *Haematococcus pluvialis*.

Fatty acid class distributions and species-level profiles also differed substantially across taxa and are presented as percentages of total fatty acids (Fig. 2c-e). Absolute fatty acid concentrations on a dry-weight basis used in the intake modeling framework are provided in Supplementary Table S6. Several microalgae were enriched in omega-3 polyunsaturated fatty acids, whereas others were dominated by saturated or omega-6 fractions. Species-level profiling highlighted elevated eicosapentaenoic acid (EPA) in *Nannochloropsis gaditana* and high docosahexaenoic acid (DHA) in *Schizochytrium limacinum*. Together, these results show that the selected edible algal biomass products differed substantially in biochemical composition, supporting product- and species-specific evaluation rather than assuming that algal biomass sources are nutritionally interchangeable.

### Trace element screening defines safety-aware intake boundaries

To evaluate these products within realistic safety bounds, we next quantified essential minerals and trace elements of toxicological relevance (Supplementary Table S3). Essential mineral concentrations varied across taxa, with relatively high iron detected in *Chlorella* sp., *Porphyridium purpureum*, and *Tetraselmis chuii*, and substantial variation was also observed for zinc and manganese.

Trace elements of toxicological relevance, including lead (Pb), total arsenic (As), and total mercury (Hg), were below detection limits in most samples. Detectable concentrations occurred in selected products, including *Dunaliella salina* (A), *Nannochloropsis salina*, and *Gigartina skottsbergii*, indicating that trace element accumulation varied across source and product type. Among detectable samples, Pb reached 2.48 ± 0.13 µg g⁻^1^ DW in *Dunaliella salina* (A) and 3.36 ± 0.35 µg g⁻^1^ DW in *Nannochloropsis salina*, whereas total As reached 16.03 ± 0.39 µg g⁻^1^ DW in *Gigartina skottsbergii*.

To interpret these values in a dietary context, conservative intake screening thresholds were estimated from health-based exposure benchmarks and used to define upper-bound intake constraints for subsequent modeling (Supplementary Table S3). Species with detectable trace elements were retained in the analysis, but intake-limiting constraints were applied where modeled exposure could exceed conservative thresholds. These data support the use of safety-aware intake constraints rather than binary exclusion of algal products from dietary consideration.

### Safety-aware modeling identifies gram-scale omega-3 contribution

**Fig. 3:**
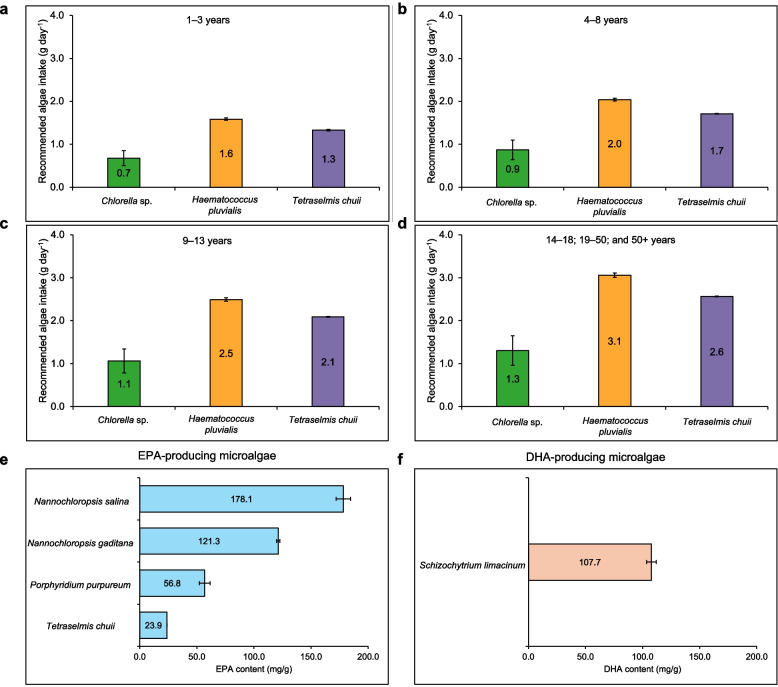
Age-dependent omega-3 contribution potential of selected microalgae based on biomass intake requirements. **a-d,** Dry biomass required for selected high-performing microalgae species to provide 20% of age-specific alpha-linolenic acid (ALA) reference intake for four age groups (1–3, 4–8, 9–13, and ≥ 14 years). Values were derived from measured fatty acid composition and total lipid content and are expressed as g dry weight day^−1^. Bars represent mean values, and error bars indicate standard deviation across measured compositions. **e,** Eicosapentaenoic acid (EPA) content of selected EPA-producing microalgae, expressed as mg g^−1^ dry weight. **f,** Docosahexaenoic acid (DHA) content of selected DHA-producing microalgae, expressed as mg g^−1^ dry weight. EPA and DHA are presented as compositional attributes and are not used as formal intake reference targets

To evaluate whether algae can contribute meaningfully to omega-3 intake within realistic dietary limits, the dry biomass required to provide 20% of age-specific alpha-linolenic acid (ALA) reference intake was calculated for each species (Fig. 3). Across species and age groups, required biomass ranged from approximately 0.7 to 3.1 g dry weight per day, depending on lipid content and fatty acid composition (Fig. 3a-d). This analysis was used as a comparative visualization of biomass efficiency and was separate from the final allocation model, which applied lower minimum ALA requirements together with EPA, DHA, biomass-cap, user-defined, and trace element-based screening constraints. Species with higher ALA concentrations required substantially lower intake levels, with several achieving 20% of age-specific intake at ~ 1 g dry biomass per day. In addition to ALA, EPA- and DHA-containing species contributed long-chain omega-3 fatty acids (Fig. 3e,f), although these are not currently included as adequacy metrics in dietary reference frameworks.

**Fig. 4:**
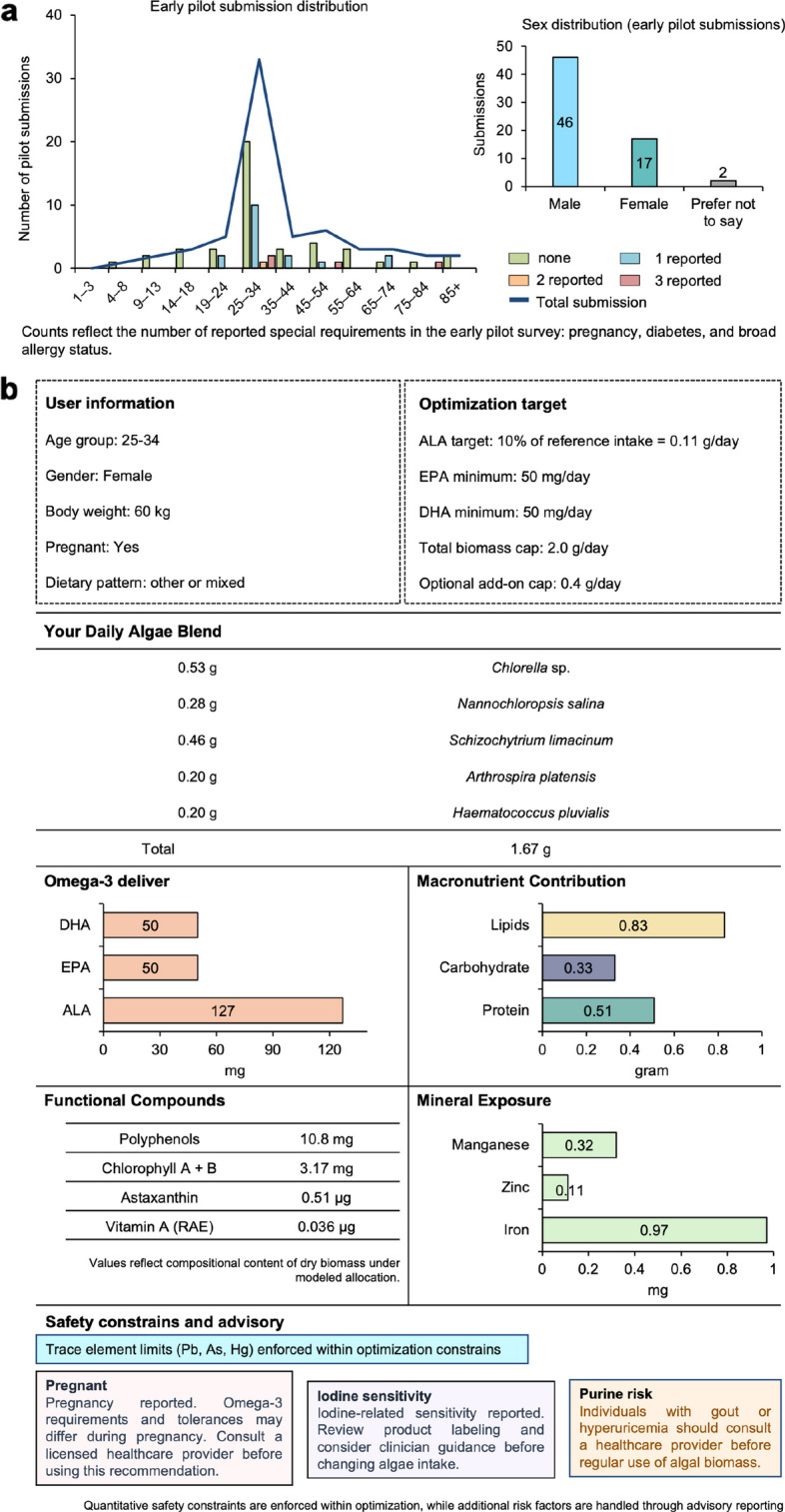
Constraint-based dietary modeling framework for algae intake under nutritional and safety constraints. **a,** Distribution of pilot submissions showing demographic characteristics and dietary or health-related considerations used to define model input ranges. Numbers indicate the count of user-reported considerations incorporated into the personalized report (e.g., pregnancy/lactation status, iodine sensitivity, allergies, medication interactions, or other user-reported restrictions). **b,** Representative modeled algae allocation based on age, sex, body weight, and dietary constraints. The model distributes biomass across species to meet omega-3 intake targets while remaining within total intake limits and trace element-based screening thresholds. Additional risk-related attributes (including pregnancy status, iodine sensitivity, and purine-associated conditions) are incorporated through a post-optimization advisory layer that does not alter model outputs. Nutritional outputs derived from modeled biomass allocation include omega-3 fatty acid delivery, macronutrient contribution, functional bioactive intake, and estimated mineral exposure, all based on experimentally measured compositional data. Colors used in the representative report panels are illustrative and do not indicate a fixed species-specific mapping across different report components. Further model structure and model structure and example outputs are provided in Supplementary Figs. S3 and S4

To assess feasibility under combined nutritional and safety constraints, a constraint-based dietary modeling framework was applied (Fig. 4; Supplementary Fig. S3 and S4). Model constraints included a total dry biomass limit of ≤ 2.0 g day⁻^1^, a minimum ALA contribution equivalent to 10% of the age-specific reference value, minimum representation targets of 50 mg EPA and 50 mg DHA where available, user-defined restrictions, and product-specific intake caps derived from trace element-based screening. Example user-facing reports generated from this framework are shown in Supplementary Fig. S3, and the workflow structure and model logic are summarized in Supplementary Fig. S4.

Under these combined constraints, multi-species allocations improved omega-3 coverage while remaining within practical intake and conservative screening thresholds (Fig. 4). Modeled outputs also indicated concurrent delivery of protein, minerals and functional compounds derived from measured compositional profiles. These analyses show that selected algae combinations can be allocated within realistic intake ranges while satisfying both nutrient contribution targets and safety-aware constraints.

### Community consumption patterns reveal limited dietary integration

**Fig. 5:**
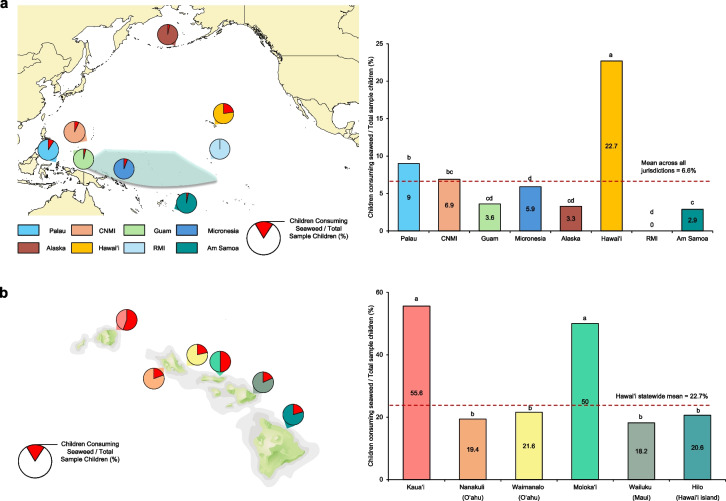
Spatial heterogeneity in seaweed consumption among Pacific Island children. **a,** Prevalence of seaweed intake across 11 Pacific Island jurisdictions. Bars represent the percentage of children reporting seaweed consumption; letters indicate statistically distinct groups (chi-square test of independence, χ.^2^ = 126.7, d.f. = 10, *P* < 0.0001; post-hoc pairwise proportion tests with Holm correction). The dashed line indicates the mean prevalence across jurisdictions (6.6%). **b,** Prevalence of seaweed intake across major Hawaiʻi islands analyzed using the same statistical approach. The dashed line indicates the statewide mean (22.7%)

To evaluate whether nutrient-dense algae currently contribute to diets, community consumption data were analyzed to assess reported seaweed-consumption prevalence among children aged 2–8 years across Pacific Island regions (Fig. 5; Supplementary Tables S5 and S6). Because portion-size data were not used to estimate nutrient contribution, this analysis was interpreted as an indicator of exposure and dietary familiarity rather than quantitative algal-derived nutrient intake.

Reported intake frequencies suggest that algae are not currently consumed at levels sufficient to contribute meaningfully to daily nutrient intake for most children, despite high compositional nutrient density (Figs. 2–4). This pattern is relevant because early dietary exposure influences food familiarity, acceptance, and long-term consumption behavior.

Together, these results indicate that algae intake in the studied populations is characterized by low frequency and limited dietary integration. Figure 5, therefore, complements compositional and modeling analyses by providing empirical evidence that dietary contribution depends on consumption behavior as well as nutrient composition.

### Food-compatible incorporation supports practical biomass translation

To evaluate whether gram-scale algae intake is compatible with realistic dietary patterns, selected algal biomass was incorporated into commonly consumed food matrices at low inclusion levels. Formulations included staple-style products such as noodles to assess whether nutritionally relevant intake levels could be achieved within typical portion sizes.

Low inclusion levels (approximately 1–2% dry weight substitution) enabled delivery of gram-scale algal biomass per serving while maintaining visible product structure and food-format feasibility (Supplementary Fig. S5). At these levels, a standard serving provided approximately 1 g dry algal biomass, consistent with intake ranges identified in the dietary modeling framework.

These results indicate that gram-scale intake levels can be achieved through incorporation into familiar food matrices rather than requiring high-volume consumption of algae as a standalone ingredient. Because algae function as nutrient-dense ingredients rather than bulk energy sources, food matrix incorporation provides a potential pathway for aligning modeled intake targets with realistic dietary behaviors.

## Discussion

Global production and market analyses indicate that algal biomass is already produced at a large scale and continues to expand, yet only a small fraction is used directly for human nutrition (Fig. 1; Supplementary Fig. S1; Supplementary Table S1), consistent with previous assessments showing that most algal biomass is directed toward non-food applications such as hydrocolloids, feed, and bioactive extracts (Araújo et al. 2021; FAO 2022, 2023, 2024). For edible algal biomass, this allocation pattern highlights an important utilization gap: production capacity and commercial growth do not necessarily translate into food-compatible nutritional applications. This is consistent with broader challenges in translating food system innovations into nutritional outcomes (Herrero et al. 2021). From an applied biotechnology perspective, this gap emphasizes the need to evaluate algal biomass not only by production potential, but also by compositional standardization, safety screening, and application-specific use scenarios, including emerging production and formulation technologies such as 3D printing-based microalgal applications. Collectively, these components should be interpreted as a screening and decision-support framework rather than a validated product-development platform (Torres-Tiji et al. 2020; Casas-Agustench et al. 2024; Gao et al. 2024; Sun et al. 2024).

By integrating compositional analysis with dietary modeling, this study shows that selected edible algal biomass products can contribute to nutrient intake at gram-scale inclusion levels (Figs. 2–4). Across species and age groups, approximately 0.7–3.1 g dry biomass was sufficient to provide 20% of age-specific alpha-linolenic acid intake, with several species achieving this level at ~ 1 g (Fig. 3). These findings indicate that edible algae can function as concentrated nutritional bioproduct resources suitable for low-level incorporation into food systems rather than as bulk dietary components. However, substantial interspecific variation in macronutrient and fatty acid composition (Fig. 2) suggests that algae are nutritionally heterogeneous resources, with species-specific roles in dietary applications. This distinction is important for algal bioproduct development because edible algae should not be treated as interchangeable biomass.

Constraint-based modeling further demonstrated that combining species improves omega-3 balance while remaining within intake and trace element-based screening limits (Fig. 4). Beyond omega-3 fatty acids, modeled allocations also contribute protein, minerals, and bioactive compounds, supporting a multi-nutrient contribution framework. The measured pigments and polyphenols provide additional information on compositional diversity among species and may be useful for product characterization and ingredient selection. However, their presence and concentration should not be interpreted as direct evidence of physiological or nutritional benefit because bioavailability, dose–response relationships, and processing stability were not evaluated in the present study. For edible algal biomass, this type of safety-aware modeling provides a practical bridge between compositional profiling and application design. Evaluating nutrient-dense foods within realistic intake constraints, therefore, provides a more meaningful assessment of dietary contribution than compositional data alone. This aligns with contemporary dietary guidelines emphasizing overall dietary patterns and food quality rather than single-nutrient optimization (U.S. Department of Agriculture & U.S. Department of Health And Human Services 2026), and with risk-based food safety frameworks that consider exposure as a function of both concentration and intake (Flannery & Middleton 2022).

Edible algae have long been recognized as nutrient-rich food sources that provide dietary fiber, essential fatty acids, minerals, and bioactive compounds, and are regularly consumed in some regions as part of traditional diets (Cherry et al. 2019). Recent work in aquaculture also shows that dietary macroalgae can alter amino acid metabolism through host-microbiome interactions, supporting the broader biological relevance of macroalgae as dietary biomass (An et al. 2025). However, community consumption data (Fig. 5) indicate that the translation of edible algal biomass into dietary use is constrained not only by composition or safety, but also by exposure, familiarity, and food context. Despite their established role in some diets, intake remains low and episodic, particularly among children, and microalgae are largely absent from routine consumption. This pattern is consistent with evidence that early-life dietary exposure strongly shapes long-term food preferences and health outcomes, and that diet quality varies across Pacific Island populations in relation to local food environments and access (Dela Cruz et al. 2023; Wilken et al. 2013).

In island and coastal food systems, where reliance on imported foods is high and local production capacity is constrained, nutrient-dense and locally cultivable foods such as algae have clear potential as regionally relevant edible biomass resources. However, the present results show that nutritional potential alone does not translate into dietary impact. Instead, the primary limitation is the lack of integration into commonly consumed and culturally familiar food formats. Consumer research on microalgae-based foods similarly shows that acceptance and willingness to pay depend on product attributes such as flavor, price, origin, production method, and packaging, reinforcing that successful adoption depends on food format and consumer context rather than nutrient value alone (Meixner et al. 2023). Although prototype noodles and desserts demonstrated that algal biomass can be incorporated into familiar food formats at low inclusion levels (Supplementary Fig. S5), sensory acceptability was not evaluated in the present study. Therefore, these examples should be interpreted as proof-of-concept demonstrations of formulation feasibility rather than evidence of consumer acceptance. Similar work has shown that microalgae-containing foods are affected by food technology neophobia, consumer segmentation, and perceived naturalness, indicating that bioproduct development must consider formulation and end-use context as well as biochemical composition (Van der Stricht et al. 2024).

By linking gram-scale nutritional feasibility (Figs. 2–3), constraint-based modeling (Fig. 4), and observed consumption behavior (Fig. 5), this study demonstrates that nutrient density, safety, and intake feasibility are necessary but not sufficient conditions for dietary impact. These findings indicate that nutrient density alone is not a good predictor of real-world dietary contribution, and that integration into familiar food systems is a critical determinant of whether nutrient-dense foods translate into meaningful improvements in population nutrition.

This study has several limitations that define the scope of interpretation. The sample set was designed for exploratory screening across edible algal biomass types and did not include multiple independent production batches for every species. Therefore, the measured values should not be interpreted as definitive species-level nutritional profiles. Future work should include multiple strains, suppliers, cultivation systems, harvest stages, processing methods, and production batches to establish validated compositional ranges for product development. Nutrient composition and trace element concentrations were measured for selected species and sources and may vary depending on cultivation conditions, environmental factors, and processing methods (Food And Agriculture Organization of the United Nations & World Health Organization 2019). The compositional assays used here were intended to support comparative screening and intake modeling rather than official nutrition-label quantification. Dietary modeling was based on measured compositional data and established reference intake values, and therefore does not explicitly incorporate variability in bioavailability, dietary interactions, food-matrix effects, or nutrient retention during processing. Community consumption data were derived from survey-reported intake and were used to evaluate reported exposure and familiarity rather than quantitative nutrient intake from seaweed. Because portion-size information and preparation practices were not consistently available across sites, these data may not fully capture variation in amount consumed, food form, or nutrient contribution.

The trace element analysis should also be interpreted as preliminary risk screening rather than safety validation. Because arsenic was measured as total arsenic, applying inorganic arsenic benchmarks to total arsenic provided a conservative upper-bound screening assumption, but does not replace arsenic speciation or refined exposure assessment. Accordingly, model outputs should be interpreted as conservative feasibility estimates rather than validated safety recommendations. In addition, iodine and cadmium were not incorporated as primary model constraints, although both can be relevant for seaweed-based products and should be included in future product-specific safety evaluations. Product-level contaminant variability across suppliers and production batches also remains an important uncertainty (Dujardin et al. 2023; Jönsson & Nordberg Karlsson 2024; Casas-Agustench et al. 2024).

In addition, although the framework provides nutrient-based intake guidance, it does not recommend specific commercial products because algal composition, quality, and safety can vary substantially across sources. Implementation would therefore require products with transparent sourcing, compositional labeling, and contaminant screening. The food-incorporation trials were intended as proof-of-concept demonstrations of food-format feasibility and were not designed to evaluate sensory acceptance, nutrient retention during processing, consumer preference, shelf-life stability, or commercial scalability. The compositional analyses were designed to support comparative screening and dietary modeling rather than comprehensive nutritional characterization. Although dietary fiber is often considered a nutritionally relevant component of edible algal biomass, fiber was not quantified directly in this study. Carbohydrates were measured as glucose equivalents for comparative compositional assessment, and therefore, species-specific fiber composition was not incorporated into the modeling framework. Despite these limitations, the integrated framework provides a conservative, constraint-based assessment of the nutritional potential of edible algal biomass under defined intake and safety-screening boundaries. Future work should extend this approach by incorporating method validation, processing-retention studies, bioavailability measurements, sensory and acceptability testing, formulation optimization, and longitudinal dietary assessment to better resolve the translation of edible algal biomass into standardized edible algal bioproduct applications.

## Conclusion

This study demonstrates that selected edible algae can provide meaningful omega-3 and multi-nutrient contributions at gram-scale intake levels when compositional value, intake feasibility, and safety constraints are considered together. Across species and age groups, approximately 0.7 to 3.1 g dry biomass was sufficient to provide 20% of age-specific ALA intake, while multi-species allocations improved long-chain omega-3 representation and contributed additional nutrients and bioactive compounds. Species differed substantially in macronutrient, fatty acid, mineral, and bioactive profiles, supporting their use as distinct nutritional biomass sources rather than interchangeable ingredients. However, community consumption data showed that algae intake remains low and episodic, particularly among children, indicating that nutrient density alone is not sufficient for dietary impact.

Collectively, these findings support the use of the proposed framework as a screening and decision-support approach for evaluating edible algal biomass within realistic nutritional and safety boundaries. Rather than serving as a validated product-development platform, the framework is intended to identify and prioritize promising edible algal biomass candidates for future nutritional bioproduct development and testing. Practical translation will require product-specific compositional validation, contaminant assessment, nutrient-retention studies, bioavailability evaluation, consumer acceptability testing, and incorporation into familiar food formats.

## Supplementary Information

Supplementary Material 1.

## Data Availability

Compositional, elemental and modeled output data are provided in the Supplementary Information. Aggregated CHL dietary data are restricted because of data-use and participant privacy requirements and are available only under appropriate approval.
