# Supplementary material for: A safety-aware intake modeling framework for translating edible algal biomass into nutritional bioproducts
Source: Adv Biotechnol (Singap). 2026 Aug 10;4(3):32. doi: 10.1007/s44307-026-00129-4 (PMC13457825; doi:10.1007/s44307-026-00129-4)
Supplement: Supplementary file 1 [file 44307_2026_129_MOESM1_ESM.pdf]

Supplementary methods

S1: Sample preparation and biomass processing

Dried algal biomass was homogenized to a fine powder prior to all analyses. For each measurement, approximately 0.10 g dry biomass was used unless otherwise specified. Sample masses ranged from 0.1000 to 0.1005 g and were treated as equivalent for calculation purposes. All results are reported on a DW basis. Powdered samples were stored in airtight containers at room temperature and protected from light until analysis. Subsamples were prepared independently for macronutrient, lipid, pigment, and elemental analyses to avoid cross-contamination. Sample preparation steps were standardized across species to ensure comparability among compositional and safety measurements.

S2: Lipid extraction and fatty-acid profiling

Total lipids were extracted from dried algal biomass using a chloroform-methanol solvent system following established protocols for microalgae and seaweed (Du et al., 2018). Approximately 0.10 g dry biomass was used per extraction. Lipid extracts were recovered gravimetrically after solvent removal and stored under inert conditions until analysis.

Extracted lipids were converted to fatty acid methyl esters (FAMEs) using acid-catalyzed transesterification. FAMEs were analyzed by gas chromatography with flame ionization detection (Agilent 8860) using a polar capillary column suitable for separation of marine fatty acids. Individual fatty acids were identified by comparison of retention times with authenticated FAME standards (37-component FAME standard; Sigma). Fatty acids were quantified as percentages of total detected fatty acids. Triglyceride class distributions were grouped into saturated fatty acids (SFA), monounsaturated fatty acids (MUFA), omega-3 polyunsaturated fatty acids (omega-3 PUFA), and omega-6 PUFA. Fatty-acid profiles and class distributions correspond to those shown in Fig. 2c and Fig. 2d. All lipid extractions and GC analyses were performed in technical triplicate for each biological replicate. Results are reported on DW basis.

S3: Protein and carbohydrates quantification

Protein content was determined using a modified Lowry assay as previously described (Slocombe et al., 2013). Dried algal biomass (5 mg) was precipitated with 0.2 mL of 25% (w/v) trichloroacetic acid at 95 °C for 15 min, followed by centrifugation (15 000 × g, 20 min). Pellets were resuspended in 0.5 mL Lowry reagent and incubated at 55 °C for 3 h prior to reaction with Folin-Ciocalteu reagent. Absorbance was measured at 750 nm using a DeNovix DS-11 FX+ spectrophotometer. Protein concentrations were quantified using bovine serum albumin standards (0-1 mg mL^{-1}) and expressed as mg protein g^{-1} DW. Total carbohydrate content was quantified using the phenol-sulfuric acid method (Dubois et al., 1956). Dried algal biomass (2 mg) was reacted with 10 µL of 80% (v/v) phenol and 1 mL concentrated sulfuric acid at 100 °C for 20 min. Absorbance was measured at 490 nm. Carbohydrate concentrations were calculated using D-glucose calibration standards (0-500 µg mL^{-1}) and expressed as glucose equivalents (mg g^{-1} DW).

S4: Pigment and polyphenol analysis

Pigments and bioactive compounds were extracted from dried algal biomass using solvent systems optimized for photosynthetic pigments and phenolics. Chlorophyll a and b were extracted in acetone:DMSO (3:2, v/v) under dark conditions and quantified spectrophotometrically at 664 and 647 nm following established equations (Porra, 2002) (Supplementary Table S4).

Carotenoids were extracted in acetone:DMSO (7:3, v/v) under dark conditions and analyzed by high-performance liquid chromatography using a reversed-phase C18 column (250 × 4.6 mm, 5 µm). Separation was achieved using a methanol-based solvent gradient, and carotenoids were detected at 455 nm. β-Carotene and astaxanthin were identified and quantified using authenticated external standards (Lourenço-Lopes et al., 2022). Additional carotenoids, including canthaxanthin, echinenone, and lycopene, were identified based on retention time matching but were not quantified. Representative chromatograms are shown in Supplementary Fig. S2.

Total polyphenols were extracted in 80% methanol and quantified using the Folin-Ciocalteu assay, with absorbance measured at 760 nm (Kolackova et al., 2020). Results were expressed as micrograms gallic acid equivalents per gram dry weight (µg GAE g^{-1} DW) using a gallic acid calibration curve ($R^2 > 0.99$) (Supplementary Table S4).

S5: Elemental analysis and heavy metal screening

Elemental composition of algal biomass was quantified by inductively coupled plasma–based analysis following acid digestion of dried samples. Approximately 0.10 g dry biomass was digested for each analysis, and elemental concentrations were calculated on a DW basis.

Trace elements of food-safety relevance, including lead (Pb), total arsenic (As), and total mercury (Hg), were quantified alongside nutritionally relevant minerals (including iron, zinc, manganese, and silicon). Instrument detection limits (IDLs) were determined for each element using laboratory blanks, defined as three times the standard deviation of repeated blank measurements. Wavelengths used for quantitation of each element; 5 ppm yttrium was used as the internal standard. Elemental concentrations below the IDL were classified as non-detects and reported as not detected.

Measured elemental concentrations represent the mean ± s.d. of biological replicates, with analytical measurements performed in technical triplicate. Quality control procedures included blank subtraction and verification against certified reference materials where available. Elemental data were used to characterize species-specific trace element profiles and to inform subsequent conservative intake modeling.

S6: Maximum screening intake

To contextualize elemental concentrations in relation to dietary exposure, conservative maximum safe intake thresholds were estimated for selected trace elements. For elements with detected concentrations, upper-bound values were defined as the mean plus two standard deviations (mean + 2 × s.d.) across biological replicates. For elements not detected, upper-bound concentrations were conservatively defined using the biomass-based instrument detection limit (IDL).

Maximum safe intake (g DW day^{-1}) was estimated by dividing applicable health-based intake benchmarks by the corresponding upper-bound elemental concentrations. Screening focused on

lead (Pb), total arsenic (As), and total mercury (Hg), which are commonly prioritized in food safety assessments.

Lead exposure was evaluated using the US Food and Drug Administration interim reference level (IRL) for children (2.2 μg day^{-1}), as defined in FDA risk assessments supporting the Closer to Zero initiative (Flannery & Middleton, 2022). Arsenic exposure was assessed using the U.S. Environmental Protection Agency reference dose for inorganic arsenic (0.3 μg kg^{-1} day^{-1}) (Agency for Toxic Substances and Disease Registry, 2007; U.S. Environmental Protection Agency IRIS Program, 2025), conservatively applied to measured total arsenic concentrations. Mercury exposure was screened using the European Food Safety Authority tolerable weekly intake for methylmercury (1.3 μg kg^{-1} body weight $week^{-1}$, expressed as mercury), converted to a daily equivalent (EFSA Panel on Contaminants in the Food Chain (CONTAM), 2012).

A conservative child body weight of 20 kg was assumed for intake screening. Resulting maximum safe intake values represent conservative screening thresholds intended to contextualize potential exposure and do not constitute dietary recommendations.

S7: Dietary reference values and age-specific nutrient modeling

Age-specific nutrient modeling was conducted to evaluate the potential contribution of edible algae to omega-3 fatty acid intake across life stages. Modeling was based on dietary reference intakes for omega-3 fatty acids defined within the US dietary framework, which specifies adequate intake (AI) values for alpha-linolenic acid (ALA) by age and sex.

For each algae species retained after elemental screening, the dry biomass intake required to provide 20% of the age-specific ALA reference intake was calculated. A 20% DV contribution was selected as a conservative partial-contribution benchmark for cross-species comparison. Notably, 20% DV corresponds to the FDA threshold for a “high” or “excellent source” labeling claim, providing regulatory context for the scale of contribution modeled (U.S. Food and Drug Administration, 2024). This parameter serves solely as a modeling benchmark and does not represent intake guidance, therapeutic recommendation, or safety threshold. Calculations were performed separately for children, adolescents, and adults using age-specific ALA reference values.

ALA concentrations were derived from fatty-acid compositional data (% of total fatty acids) and total lipid content, and expressed on a DW basis. Required intake (g DW day^{-1}) was calculated by dividing the target ALA amount (20% of the age-specific reference intake) by the species-specific ALA concentration.

Although several algal products contained appreciable amounts of long-chain omega-3 fatty acids, including EPA and DHA, no formal dietary reference intakes exist for EPA or DHA within the US dietary reference intake framework. EPA and DHA were therefore not used as primary adequacy targets in the age-specific intake calculations shown in Fig. 3. However, in the final allocation model (Supplementary Method S8), EPA and DHA were incorporated as secondary representation targets to encourage inclusion of long-chain omega-3 sources when feasible within the overall biomass and safety-screening constraints. These values were used as practical allocation targets and were not interpreted as clinical intake recommendations.

At modeled intake levels, contributions of additional nutrients, including protein, carbohydrates, minerals, and bioactive compounds, were calculated based on measured compositional profiles. These nutrients were treated as complementary outputs and were not used as primary drivers of intake modeling.

S8: Constraint-based dietary modeling framework

A constraint-based dietary modeling framework was developed to evaluate how edible algae biomass can contribute to nutrient intake under realistic dietary and safety constraints. The model converts compositional data into daily intake allocations by solving a constrained allocation problem in which biomass from multiple algae species is combined to meet nutrient contribution targets while remaining within practical intake limits and conservative safety thresholds.

Model inputs included species-specific compositional data measured in this study, including lipid content, fatty acid composition, macronutrient composition, bioactive compounds, and trace element concentrations. Dietary reference intake values for alpha-linolenic acid (ALA) were used as the primary adequacy target, as ALA is the omega-3 fatty acid with established dietary reference intake values within the US dietary framework. Age group, sex, and body weight were used to select the applicable ALA reference intake and to apply bounded anthropometric scaling to the modeled ALA contribution only. Body-weight scaling was limited to ±20% relative to reference median body weights to maintain comparability with dietary reference intake frameworks and to avoid clinical weight-based dosing interpretation (Fryar et al., 2021; U.S. Centers for Disease Control and Prevention, 2024).

The decision variables in the model represent daily dry biomass intake from each algae species:

$$x_i = \text{ daily intake of species } i \ (g\ day^{-1})$$

Total daily algae intake is:

$$X = \sum_i x_i$$

The model includes multiple constraints representing realistic dietary intake limits, nutrient contribution targets, and safety screening thresholds.

Total biomass constraint:
Total daily algae intake was constrained to a maximum practical intake level:

$$\sum_i (x_i) \leq 2.0 \ (g\ day^{-1})$$

This constraint represents realistic intake achievable through incorporation of algae into commonly consumed foods at low inclusion levels.

Omega-3 adequacy constraint (ALA):
ALA intake from the biomass allocation must meet a defined fraction of the age- and sex-specific ALA reference intake:

$$\sum_i (x_i * ALA_i) \geq f * ALA_{ref}$$

Where ALA_i is the ALA concentration of species i,
ALA_{ref} is the age- and sex-specific ALA reference intake,
f is the target contribution fraction.
This threshold was used as the practical minimum contribution target in the final allocation model. This differs from the 20% ALA benchmark used in Fig. 3, which was used only for visualization and cross-product comparison.

Long-chain omega-3 structural constraints:
These thresholds were included as practical long-chain omega-3 representation targets and were not interpreted as formal DRI-based adequacy targets.

To ensure representation of long-chain omega-3 fatty acids, minimum contribution constraints were applied for EPA and DHA:

$$\sum_i (x_i * EPA_i) \geq EPA_{min}$$

$$\sum_i (x_i * DHA_i) \geq DHA_{min}$$

Trace element safety constraints:
For species with detectable trace element concentrations, maximum allowable intake was constrained based on health-based
exposure thresholds:

$$\frac{(C_{ij} \times x_i)}{BW} \leq RfDj$$

Where C_{ij} is the concentration of trace element *j* in species *i* (μg g^{-1} DW),
x_i = intake of species *i* (g day^{-1}),
BW is body weight,
RfDj is the reference dose for trace element *j*.

Objective function:
The model minimizes total daily algae biomass while satisfying all constraints:

$$Minimize \sum_i xi$$

Model outputs include daily biomass allocation by species, total omega-3 contribution (ALA, EPA, DHA), macronutrient contribution, bioactive compound contribution, and estimated trace element exposure.

Additional user inputs including pregnancy or lactation status, health conditions, medication use, allergy status, dietary pattern, and product quality information were used to generate advisory messaging and contextual reporting but did not modify core nutritional constraints or safety thresholds. This structure ensures that the model provides conservative nutritional guidance without functioning as a clinical decision tool.

S9: Advisory layers for safety and reporting

S9.1: Iodine advisory rationale and implementation

Iodine was incorporated as a contextual safety advisory layer because it is essential for thyroid hormone synthesis and has a relatively narrow margin between recommended intake and upper intake levels across life stages. In U.S. dietary guidance, iodine requirements and tolerable upper intake levels differ by age and reproductive status, with lower upper limits in children, making iodine an important consideration for personalized reporting (National Institutes of Health, Office of Dietary Supplements, 2024).

Iodine was not implemented as a quantitative optimization constraint because iodine concentrations were not measured for all candidate products and are known to vary substantially across algae taxa, product forms, and sourcing conditions. This variability is well documented in commercial edible algae surveys and regulatory exposure assessments, which show wide differences in iodine concentrations and exposure risk depending on species and product characteristics (Casas-Agustench et al., 2024; Dujardin et al., 2023).

Accordingly, iodine was handled through a conservative advisory framework rather than as a nutrient allocation constraint. In the current implementation, user-reported thyroid disorder, thyroid medication use, pregnancy, or lactation activate iodine-related report messaging, including label review prompts and recommendations to prioritize products with transparent sourcing and, where available, product-specific compositional testing. This advisory layer does not modify the optimization constraints or objective function of the dietary allocation model.

S9.2: Nucleic acid and purine advisory rationale and implementation

Potential nucleic acid and purine burden was incorporated as a precautionary advisory layer because some microalgal biomasses contain substantial nucleic acid content, and excessive intake of nucleic acid-rich biomass may increase uric acid burden in susceptible individuals. Recent human feeding studies further support this concern by showing elevated postprandial serum uric acid concentrations following ingestion of microalgal biomass preparations, including Spirulina and *Chlorella*, relative to control conditions (Williamson et al., 2024).

This factor was not implemented as a quantitative optimization constraint because nucleic acid and purine contents were not measured consistently across all candidate species and product formats included in the present dataset. Instead, gout or hyperuricemia status was treated as a health-flag trigger within the advisory layer. In the current architecture, users reporting gout or hyperuricemia receive clinician-referral language and additional caution messaging, with stronger wording applied when the intended product form is whole biomass, powder, or tablet, or when planned use is daily for at least 3 months.

This advisory framework supports conservative risk communication while preserving the compositional comparability and mathematical structure of the omega-3 optimization model.

S10: Community algae consumption survey

Community-level algae consumption patterns were assessed using existing dietary survey data from the Children's Healthy Living (CHL) Program collected between 2013 and 2019. The dataset included 6055 children aged 2-8 years across Pacific Island jurisdictions. Dietary intake was reported by parents using validated questionnaires that included a specific item for seaweed (limu) consumption (Supplementary Table S5 and S6). Aggregate data describing the proportion of children reporting any seaweed intake were summarized by region and survey year using R (v4.2.2). The CHL Program was approved by the University of Hawaiʻi Human Studies Program (protocol CHL-2012-003). Only de-identified, aggregated data were analyzed. The analysis was deemed not human subjects research under University of Hawaiʻi IRB exemption criteria (IRB Protocol ID: 2026-00185).

S11: Community-based product development

To assess practical feasibility of algae incorporation into local foods, prototype algae-enriched products were developed in collaboration with a local food enterprise (Adela's Country Eatery, Oʻahu) and University of Hawaiʻi CTAHR extension specialists. Two product formats were prepared: algae-infused noodles (Fig. S4c, S4d, S4e) and algae-enriched desserts (Fig. S4f). *Arthrospira platensis* and *Chlorella* sp. were incorporated at 1-5% (w/w) of base formulation to achieve modeled nutrient targets, including ≥20% of age-specific omega-3 adequacy or ≥10% of daily protein contribution. Final formulations included 2% *Arthrospira platensis* for noodles and 1-2 g *Arthrospira platensis* or *Chlorella* sp. per 150 g dessert serving.

Pilot batches (~100 g) were prepared in a certified kitchen following Hawaiʻi Department of Health food safety standards. Qualitative feedback on appearance, texture, and flavor was obtained from 10-15 adult participants, including restaurant staff and volunteers. No formal sensory evaluation or quantitative scoring was conducted. Activities were classified as community outreach and did not require institutional review board approval. Participants provided verbal consent and were informed of all ingredients. Final recipes and nutrient estimates were documented for educational and outreach purposes.

References

Agency for Toxic Substances and Disease Registry. (2007). *Toxicological Profile for Arsenic* (No. NBK591644). U.S. Department of Health and Human Services. https://www.ncbi.nlm.nih.gov/books/NBK591644/

Casas-Agustench, P., Hayter, J. M., Ng, O. S. B., Hallewell, L. V., Clark, N. J., & Bescos, R. (2024). Nitrate, nitrite, and iodine concentrations in commercial edible Algae: An observational study. *Foods*, *13*(16), 2615. https://doi.org/10.3390/foods13162615

Du, Z. Y., Alvaro, J., Hyden, B., Zienkiewicz, K., Benning, N., Zienkiewicz, A., Bonito, G., & Benning, C. (2018). Enhancing oil production and harvest by combining the marine alga Nannochloropsis oceanica and the oleaginous fungus Mortierella elongata. *Biotechnology for Biofuels*, *11*, 174. https://doi.org/10.1186/s13068-018-1172-2

Dubois, M., Gilles, K. A., Hamilton, J. K., Rebers, P. A., & Smith, F. (1956). Colorimetric Method for Determination of Sugars and Related Substances. *Analytical Chemistry*, *28*(3), 350–356. https://doi.org/https://doi.org/10.1021/ac60111a017

Dujardin, B., Ferreira de Sousa, R., & Gómez Ruiz, J. Á. (2023). Dietary exposure to heavy metals and iodine intake via consumption of seaweeds and halophytes in the European population. *EFSA Journal*, *21*(1), e07798. https://doi.org/10.2903/J.EFSA.2023.7798

EFSA Panel on Contaminants in the Food Chain (CONTAM). (2012). Scientific Opinion on the risk for public health related to the presence of mercury and methylmercury in food. *EFSA Journal*, *10*(12), 2985. https://doi.org/10.2903/j.efsa.2012.2985

Flannery, B. M., & Middleton, K. B. (2022). Updated interim reference levels for dietary lead to support FDA's Closer to Zero action plan. *Regulatory Toxicology and Pharmacology*, *133*, 105202. https://doi.org/10.1016/j.yrtph.2022.105202

Fryar, C. D., Carroll, M. D., Gu, Q., Afful, J., & Ogden, C. L. (2021). *Anthropometric Reference Data for Children and Adults: United States, 2015–2018* (No. 46; Vital Health Stat. Series 3, No. 46, pp. 1–44). National Center for Health Statistics. https://stacks.cdc.gov/view/cdc/100478

Kolackova, M., Chaloupsky, P., Cernei, N., Klejdus, B., Huska, D., & Adam, V. (2020). Lycorine and UV-C stimulate phenolic secondary metabolites production and miRNA expression in *Chlamydomonas reinhardtii*. *Journal of Hazardous Materials*, *391*, 122088. https://doi.org/10.1016/j.jhazmat.2020.122088

Lourenço-Lopes, C., Fraga-Corral, M., Garcia-Perez, P., Carreira-Casais, A., Silva, A., Simal-Gandara, J., & Prieto, M. A. (2022). A HPLC-DAD method for identifying and estimating the content of fucoxanthin, β-carotene and chlorophyll a in brown algal extracts. *Food Chemistry Advances*, *1*. http://hdl.handle.net/10400.22/21861

National Institutes of Health, Office of Dietary Supplements. (2024). *National Institutes of Health, Office of Dietary Supplements* [Government website]. Iodine Fact Sheet for Consumers. https://ods.od.nih.gov/factsheets/Iodine-Consumer/

Porra, R. J. (2002). The chequered history of the development and use of simultaneous equations for the accurate determination of chlorophylls a and b. *Photosynthesis Research*, *73*(1–3), 149–156. https://doi.org/10.1023/A:1020470224740/METRICS

Slocombe, S. P., Ross, M., Thomas, N., McNeill, S., & Stanley, M. S. (2013). A rapid and general method for measurement of protein in micro-algal biomass. *Bioresource Technology*, *129*, 51–57. https://doi.org/10.1016/J.BIORTECH.2012.10.163

U.S. Centers for Disease Control and Prevention. (2024, October 15). *CDC Growth Chart Data Files*. U.S. Department of Health and Human Services. https://www.cdc.gov/growthcharts/cdc-data-files.htm

U.S. Environmental Protection Agency IRIS Program. (2025, January 10). *Toxicological Review of Inorganic Arsenic (CASRN 7440-38-2). Final Report.* [Reports and Assessments]. U.S. Environmental Protection Agency. https://iris.epa.gov/document/&deid=363892

U.S. Food and Drug Administration. (2024, March 5). *Daily Value on the Nutrition and Supplement Facts Labels*. FDA. https://www.fda.gov/food/nutrition-facts-label/daily-value-nutrition-and-supplement-facts-labels

Williamson, E., Monteyne, A. J., Van der Heijden, I., Abdelrahman, D. R., Murton, A. J., Hankamer, B., Stephens, F. B., & Wall, B. T. (2024). Ingestion of ‘whole cell’ or ‘split cell’ Chlorella sp., Arthrospira sp., and milk protein show divergent postprandial plasma amino acid responses with similar postprandial blood glucose control in humans. *Frontiers in Nutrition*, *11*, 1487778. https://doi.org/10.3389/fnut.2024.1487778

Supplementary Figure S1. Global patent activity related to seaweed and microalgae, 2010-2024.

Supplementary Figure S2. Representative HPLC chromatograms of carotenoid extracts from selected microalgae and seaweed species detected at 455 nm.

Supplementary Figure S3. Example of recommendation reports

Supplementary Figure S4. Workflow of the constraint-based dietary modeling framework.

Supplementary Fig. S5. Community implementation and algae-enriched food development.

Supplementary Table S1. Source data and assumptions underlying global algae production, market, publication and downstream application analyses.

Supplementary Table S2. Sources and product characteristics of edible algae used in this study.

Supplementary Table S3. Heavy metal and essential mineral composition of selected microalgae and seaweed species and associated safety screening metrics.

Supplementary Table S4. Number of children reporting consumption of seaweed or seaweed-containing food items across all CHL survey jurisdictions in 2013, 2015 and 2019.

Supplementary Table S5. Number of children reporting consumption of seaweed or seaweed-containing food items across Hawai‘i communities in 2013, 2015 and 2019.

Supplementary Table S6. Full compositional profile of selected microalgae and seaweed species used for dietary modeling.

a b

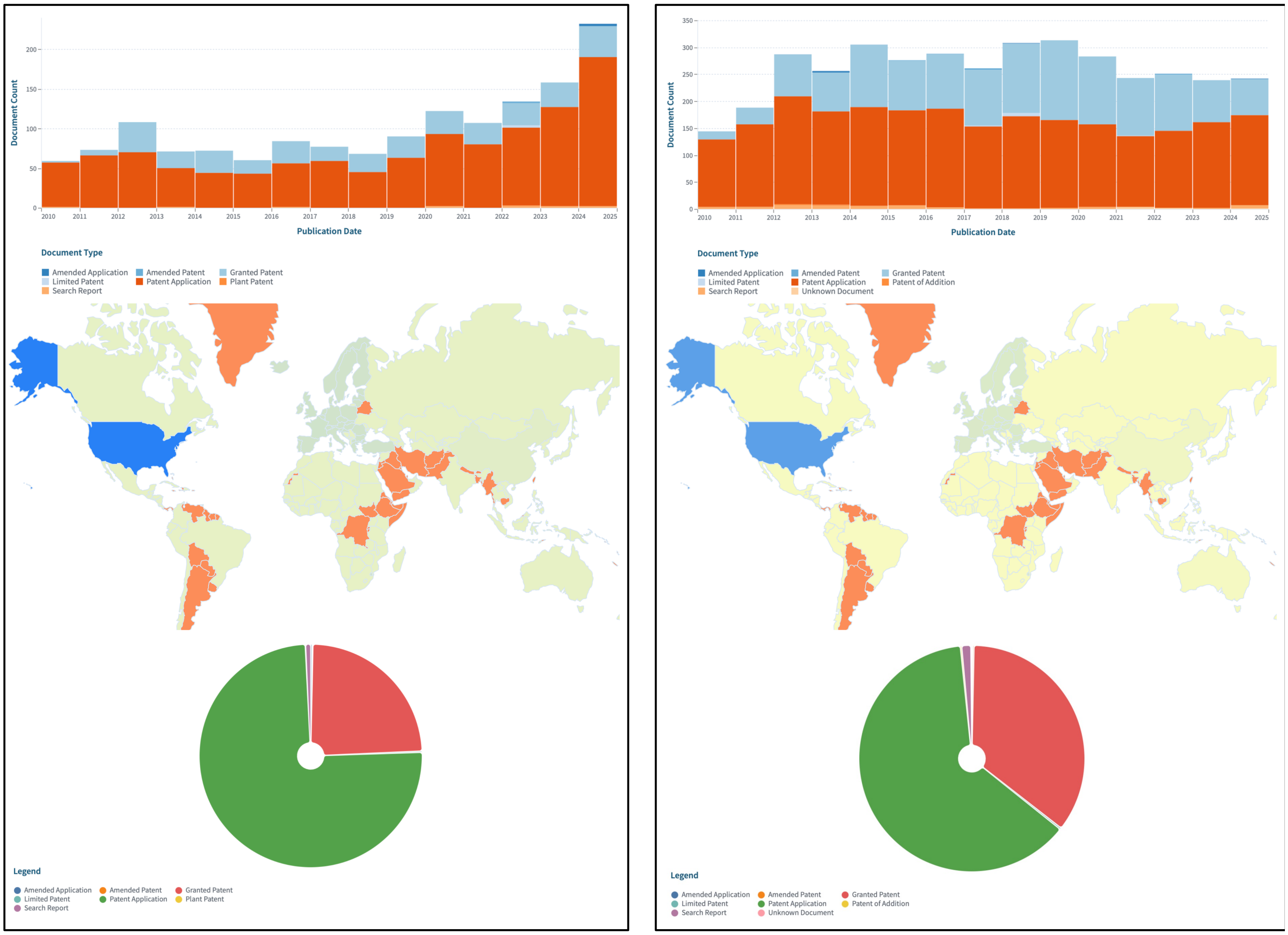

Supplementary Figure S1. Global patent activity related to seaweed and microalgae, 2010-2024.

a, Seaweed-related patents and applications, including annual document counts (top), jurisdictional distribution (middle), and document-type composition (bottom). **b**, Microalgae-related patents and applications shown using the same three panels. Patent data were retrieved from Lens.org (accessed 18 November 2025) using the queries “seaweed OR macroalgae” and “microalgae OR microalgal”, restricted to 2010-2024 and including all jurisdictions and all document types. Counts correspond to individual patent documents (not consolidated patent families).

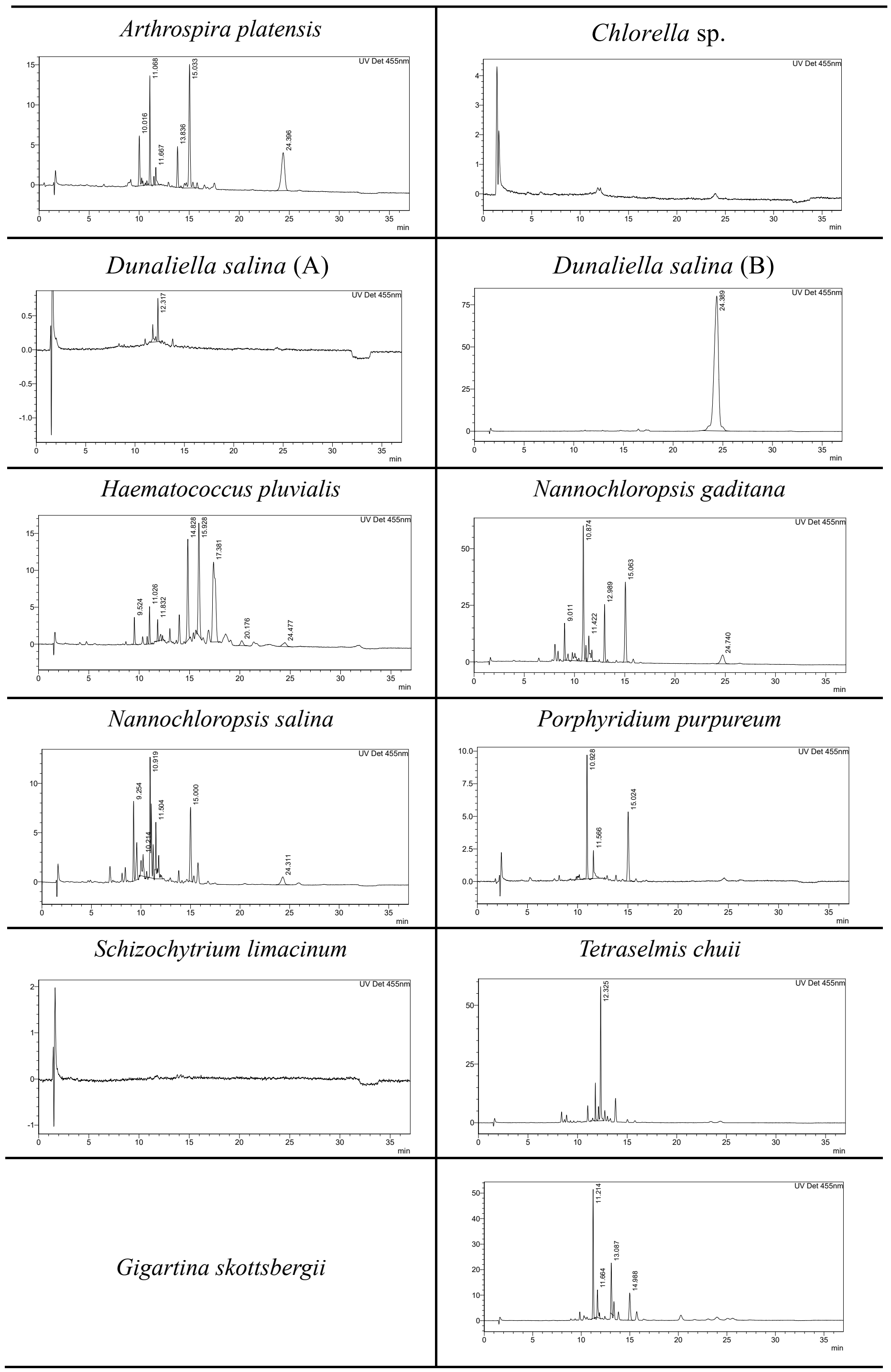

Supplementary Fig. S2. Representative HPLC chromatograms of carotenoid extracts from selected microalgae and seaweed species detected at 455 nm.

Panels show chromatograms for *Arthrospira platensis*, *Chlorella* sp., *Dunaliella salina* (A), *Dunaliella salina* (B), *Haematococcus pluvialis*, *Nannochloropsis gaditana*, *Nannochloropsis salina*, *Porphyridium purpureum*, *Schizochytrium limacinum*, *Tetraselmis chuii* and *Gigartina skottsbergii*. Major peaks correspond to astaxanthin (approximately 9 to 10 min) and β-carotene (approximately 25 min), with additional peaks assigned to canthaxanthin, echinenone and lycopene based on retention times. Astaxanthin and β-carotene were quantified using external standards. This figure underlies the carotenoid composition shown in Fig. 2b.

SeaGreen Daily Report

SeaGreen_v1.0

A personalized report that explains your modeled algae blend, what it covers, and how to use it in a practical way.

Report code: 0004 | **Generated:** 2026-04-02 16:57:26 UTC | **Retrieve later:** use these 4 digits on the home page

Full report ID: SGD-20260402-0004

REPORT SUMMARY

Your profile

Age group	**35–44**
Sex	**Female**
Body weight	**50.0 kg**
Dietary pattern	**Other or mixed**
Activity level	**Sedentary, Rarely or never exercise**

Omega-3 fatty acids are important nutrients that support heart, brain and overall health. This report estimates how algae can help meet daily omega-3 needs.

Modeled targets

ALA target ?	**0.09 g/day**
EPA minimum ?	**50.0 mg/day**
DHA minimum ?	**50.0 mg/day**
Total biomass cap ?	**2.0 g/day**

These personalized targets are estimated from your profile and used to design your microalgae blend. with body weight adjustment applied when appropriate.

EPA MINIMUM
100% met
Modeled EPA minimum

DHA MINIMUM
100% met
Modeled DHA minimum

ALA SUPPORT
100% of target
10% DV-based modeled target

BLEND STATUS
Full model fit
Within daily biomass cap

BLEND SUMMARY

Quick blend overview

Total daily blend	**1.17 g**
Chlorella sp.	**0.43 g**
Nannochloropsis salina	**0.28 g**
Schizochytrium limacinum	**0.46 g**

YOUR DAILY ALGAE BLEND

BLEND SUMMARY

Your daily microalgae blend

Chlorella sp.	**0.43 g**
Nannochloropsis salina	**0.28 g**
Schizochytrium limacinum	**0.46 g**
Total	**1.17 g**

Chlorella sp. · 36.4%
Nannochloropsis salina · 24.0%
Schizochytrium limacinum · 39.7%

COVERAGE

Omega-3 intake from your personalized algae blend

ALA **88.0 mg**
Target 88.0 mg/day · 100.0% met

EPA **50.0 mg**
Target 50.0 mg/day · 100.0% met

DHA **50.0 mg**
Target 50.0 mg/day · 100.0% met

WHY THIS BLEND

Why this blend was chosen

- The blend was selected to meet the modeled omega 3 targets while staying within the daily biomass and safety limits.
- Chlorella sp. was included to help support ALA delivery and provide a broad whole-biomass base.
- Nannochloropsis salina was included as a strong EPA-contributing species.
- Schizochytrium limacinum was included as the main DHA-contributing species in the powder blend.

WHAT IT COVERS

What this blend covers

- Meets the modeled ALA target.
- Meets the modeled EPA minimum.
- Meets the modeled DHA minimum.
- Stays within the total daily biomass cap of 2.0 g/day.
- The strict optimization mode fit within the available cap without needing fallback mode.

NUTRITION

Functional compounds

Polyphenols	**8.74 mg**
Chlorophyll A + B	**2.00 mg**
Astaxanthin	**0.000 mg**
Vitamin A (RAE)	**0.003 µg**

NUTRITION

Macronutrients

Nutrient	Amount
Protein	0.35 g
Lipids	0.19 g
Carbohydrate	0.00 g

NUTRITION

Minerals

Mineral	Amount
Iron	0.719 mg
Zinc	0.098 mg
Manganese	0.308 mg

HOW TO USE IT

USE PLAN

Daily use plan

- Total daily blend: 1.17 g/day.
- Use once daily or split into two smaller servings if that is easier for taste or routine.
- Take with food or mix into a meal or drink rather than using it dry.
- If this is your first time using algae powders, consider starting with a partial amount for 3 to 5 days before moving to the full blend.

TASTE

Taste and texture tips

- Smoothies, yogurt, oatmeal, soups, and strongly flavored sauces may help mask grassy or marine flavors.
- Citrus, berry, cocoa, ginger, cinnamon, or vanilla based foods often pair better with algae powders than plain water.
- Chlorella and Arthrospira may taste grassy or earthy, so they are often easier to use in smoothie or savory formats.
- Schizochytrium may contribute a stronger marine note, so mixing it into more flavorful foods is often preferable.

STORAGE

Storage and handling

- Store powders in a cool, dry place away from heat, humidity, and direct light.
- Keep containers tightly sealed after opening and follow the labeled expiration date and lot information.
- If the product label recommends refrigeration after opening, follow the label instructions rather than room-temperature storage.

HOW TO USE

How to use this blend

- Use this recommendation as a daily powder blend, not as an oil based product.
- The full amount can be mixed into smoothies, yogurt, oatmeal, or another food or drink that masks the taste well.
- If preferred, split the daily amount into two smaller servings taken at different times of day.
- For first time use, consider starting with a smaller portion for several days to assess tolerance before moving to the full amount.
- USDA MyPlate Kitchen recipe collection
- American Heart Association healthy eating guidance
- NIH Office of Dietary Supplements consumer fact sheet on omega-3 fatty acids

COMMON QUESTIONS

Common questions

▼ Can I take the whole blend at once?

Yes. Most users can take the full daily blend at one time with food. Splitting the blend into two servings may improve tolerance if you are new to algae powders.

▶ Can I mix the powders into food instead of water?

▶ Can I use capsules instead of loose powder?

▶ What if I already take another omega-3 supplement?

SAFETY AND QUALITY

BEFORE YOU BUY

Product quality checklist

- Match the product label to the exact species names listed in this report whenever possible.
- Choose whole algae powder or dried biomass powder. This report is not designed for oil-only products or softgels.
- Prefer third-party contaminant testing or a certificate of analysis that includes heavy metals and microbial quality.
- Check grams per serving, lot number, expiration date, and storage instructions before purchase.
- Confirm that Nannochloropsis salina is printed clearly on the label because availability varies across brands.
- If Schizochytrium limacinum is part of the blend, stay with powder or dried biomass products if you want to follow this report closely.

BUILD YOUR OWN BLEND

If preparing your own blend

- If preparing your own blend, match the exact species names listed above and combine the daily gram amounts shown in the blend table.
- Use a digital scale whenever possible because powder density varies by species and by product.
- If using capsules, convert the labeled grams per serving to the daily gram amounts shown in this report before combining products.

CLINICIAN REVIEW

Who should review this with a clinician

- No additional clinician review prompts were triggered by the answers entered for this report.

HELPFUL NOTES

Additional model notes

- Body weight scaling was applied to the ALA target.

BACKGROUND AND SUPPORT

BACKGROUND

Background references and links

This report is based on the SeaGreen Daily compositional framework and supporting manuscript materials prepared by the study team.

- NIH Office of Dietary Supplements: Omega-3 Fatty Acids Fact Sheet for Health Professionals
- FDA small entity compliance guide for ALA, EPA, and DHA nutrient content claims
- Dietary Guidelines for Americans, 2020-2025
- CDC nutrition guidance

REPORT BASIS

What this report is designed to do

Included

- Personalized blend formula
- Omega-3 target framing
- Species-specific safety limits
- Practical powder-use guidance

Not included

- Medical diagnosis
- Disease treatment advice
- Brand-specific endorsements
- Drug-dosing recommendations

Dataset SeaGreen_v1.0 · Omega 3 composition omega3_composition_v1.1 · Heavy metal caps heavy_metal_caps_v1.1 · Safety metadata species_safety_metadata_v1.0

This report provides informational nutrition guidance based on compositional modeling. It does not provide medical diagnosis, treatment, or disease specific dosing.

SHARE FEEDBACK

Help us improve future reports

Your feedback helps us improve the clarity and usefulness of SeaGreen Daily. Contact details are optional and will only be used if you request follow-up about your feedback.

How helpful was this report?

Very helpful

Would you like us to contact you about your feedback?

No

What was clear or useful?

Optional

What was confusing or missing?

Optional

Submit feedback

Supplementary Figure S3. Example of recommendation report

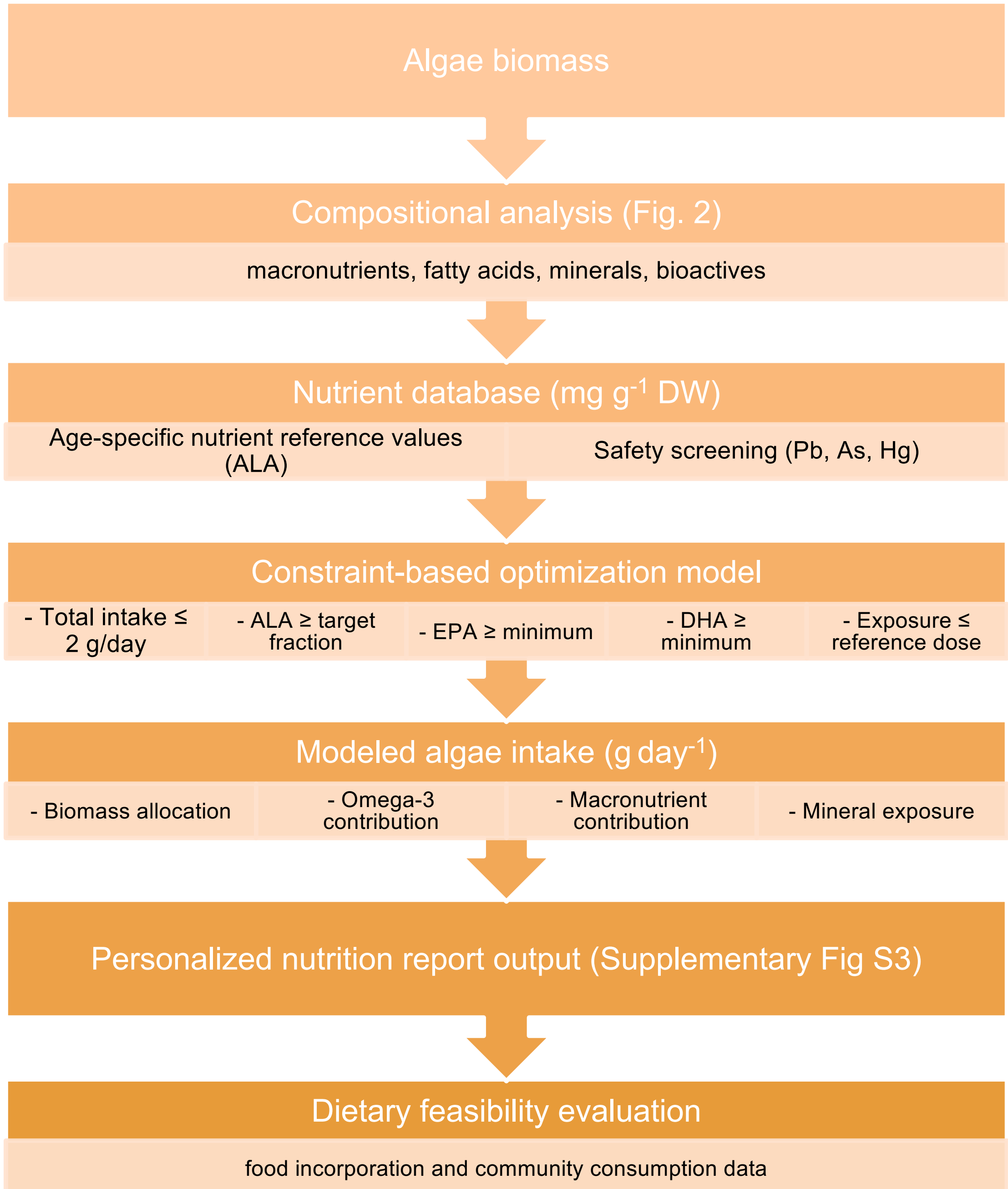

Supplementary Fig. S4. Workflow of the constraint-based dietary modeling framework.

Measured compositional data and trace element concentrations were combined with age-specific dietary reference intake values to define nutritional targets and safety constraints. Biomass allocation across species was determined by solving a constrained optimization problem that minimized total biomass while meeting omega-3 contribution targets and safety limits. Model outputs included biomass allocation, omega-3 contribution, macronutrient contribution, and estimated trace element exposure. Modeled intake levels were further evaluated in the context of food incorporation and community consumption data to assess dietary feasibility.

a b c d e f

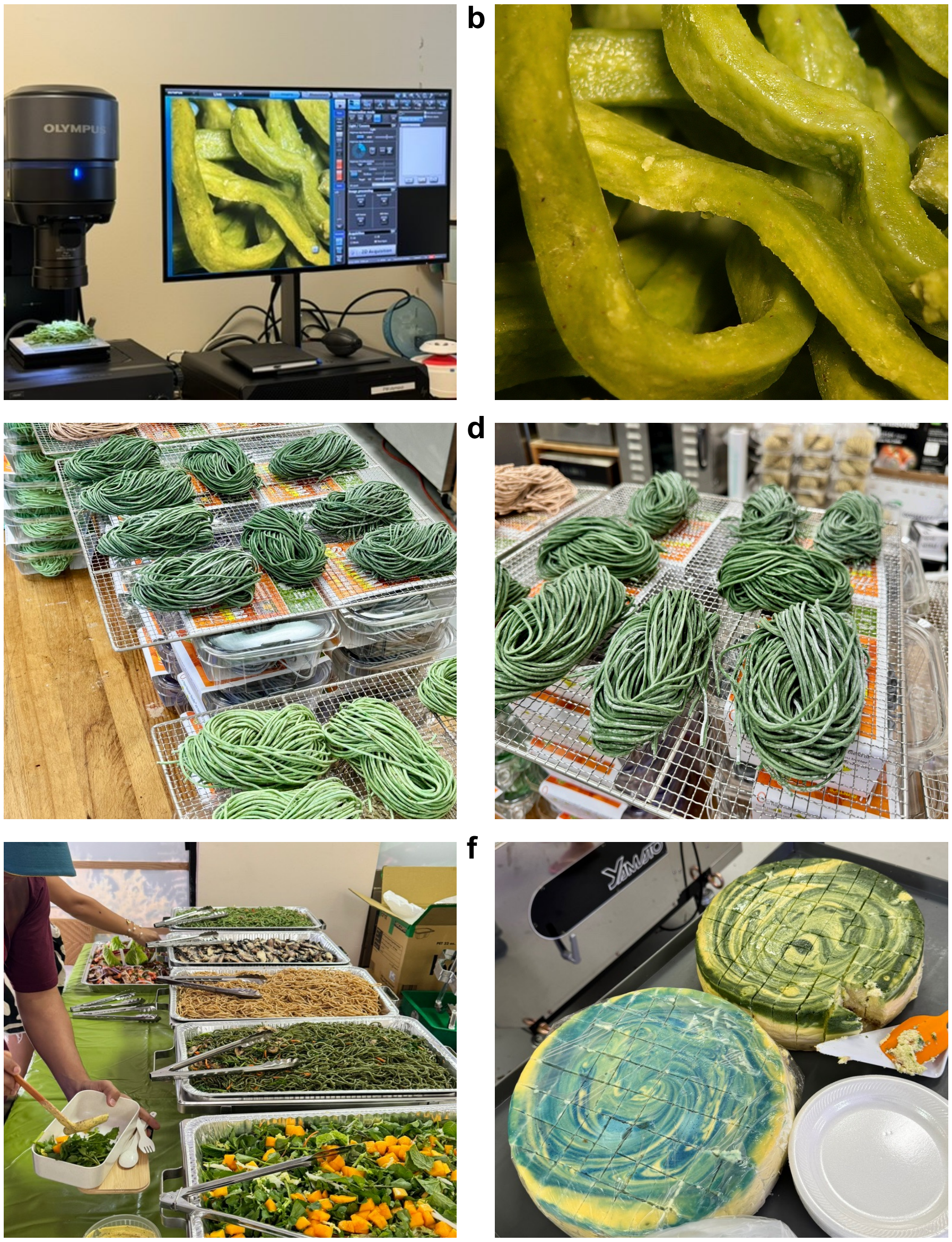

Supplementary Figure S5. Community implementation and algae-enriched food development. **a**, Digital microscopy imaging of *Chlorella*-enriched noodle surface texture using an Olympus VHX system. **b**, Close-up of *Chlorella*-enriched noodles showing characteristic green coloration. **c-e**, Production workflow at Adela's Country Eatery, Hawai‘i, illustrating dough drying, and packaging of *Chlorella*-enriched noodles. **e**, Public tasting and outreach event featuring algae-based dishes and educational materials on sustainable local foods. **f**, Spirulina- and *Chlorella*-based cheesecakes formulated as prototype algae-enriched desserts.

Supplementary Table S1. Source data and assumptions underlying global algae production, market, publication and downstream application analyses.

a, Country-level macroalgae production and estimated microalgae or algae market value underlying Fig. 1a. Macroalgae production is reported as FAO mean annual output for 2020-2022 in million tonnes wet weight. Microalgae or algae market values represent national estimates for 2024 in USD billion. **b**, Data sources, time periods and harmonized categories used for Fig. 1b, Fig. 1c and Supplementary Fig. S1. Downstream application proportions represent synthesized estimates from market assessments and review literature rather than direct measurements.

a. Country-level macroalgae production and estimated microalgae or algae market value underlying Fig. 1a.

Region / Country	Macroalgae production (Mt, wet, FAO mean 2020-2022)	Microalgae / algae market value (USD B, 2024 est.)	Notes
China	21	—	Farmed
Indonesia	9	—	Farmed
South Korea	1.8	—	Farmed
Philippines	1.4	—	Farmed
Japan	0.6	<0.1B	Farmed
Malaysia	0.4	—	Farmed
Vietnam	0.3	—	Farmed
India	0.1	<0.1B	Farmed + wild
Chile	0.1	—	Wild harvest
Peru	0.08	—	Wild harvest
Morocco	0.06	—	Wild harvest
France	0.05	<0.1B	Wild + small farms
Spain	0.03	<0.1B	Wild harvest
Portugal	0.02	—	Wild harvest
Ireland	0.02	—	Wild
Norway / UK	0.03	<0.1B	Wild + farm emerging
Thailand	0.015	—	Farmed
Myanmar	0.008	—	Farmed
United States	0.05	0.33B	Small farms
Canada	0.014	<0.1B	Wild
Russia	0.04	—	Wild harvest
Tanzania (Zanzibar)	0.103	—	Farmed
Madagascar	0.011	—	Farmed
Mozambique	0.003	—	Farmed
South Africa	0.001	<0.1B	Wild
New Zealand	<0.05	<0.1B	Wild
Australia	0.03	0.25B	Limited farms
Pacific Islands / Hawai‘i	0.01	<0.1B	Cultural
Other countries (aggregated)	0.7	—	Not mapped individually

b. Data sources and harmonized categories used for Fig. 1b and Fig. 1c

Figure panel	Data represented	Time period	Source type	Main sources	Harmonization / notes
Fig. 1b	Global seaweed market size	2020-2022	Industry market estimates	Market reports; FAO context	Values harmonized to USD billion
Fig. 1b	Global microalgae market size	2024	Industry market estimates	Market reports	Values harmonized to USD billion
Fig. 1b	Seaweed publication output	2010-2024	Web of Science	Query: "seaweed OR macroalgae"	Annual publication output
Fig. 1b	Microalgae publication output	2010-2024	Web of Science	Query: "microalgae OR microalgal"	Annual publication output
Fig. 1c	Seaweed downstream allocation	2024 estimate	Market assessment and review synthesis	FAO; review literature; market reports	Categories harmonized into human nutrition, food ingredients, feed/aquafeed, cosmetics/specialty chemicals, biofuels/bioplastics
Fig. 1c	Microalgae downstream allocation	2024 estimate	Market assessment and review synthesis	FAO; review literature; market reports	Categories harmonized into food ingredients/hydrocolloids, food whole, animal feed/fertilizers, cosmetics/nutraceuticals, biofuels/bioplastics
Supplementary Fig. S1	Patent activity	2010-2024	Lens.org	Queries: "seaweed OR macroalgae"; "microalgae OR microalgal"	Individual patent documents, not patent families

Supplementary Table S2. Sources and product characteristics of edible algae used in this study. Algal biomass was obtained from commercially available products and supplier-provided samples representing species commonly used or proposed for human consumption. Product form and source are provided to contextualize real-world availability. Where applicable, price is reported as cost per gram of dry biomass at the time of acquisition. Samples provided by suppliers are indicated as not commercially priced.

**Species (strain-neutral label)**	**Taxonomic group**	**Product form**	**Source (supplier or provider)**	**Price (USD g⁻¹ DW)**	**Note**
Arthrospira platensis	Microalgae	Powder	Online retail supplier	0.063	Retail product
Chlorella sp.	Microalgae	Powder	Online retail supplier	0.057	Retail product
Dunaliella salina (A)	Microalgae	Powder	ALGANEX	Not commercially priced	Provided for research purposes
Dunaliella salina (B)	Microalgae	Powder	Online retail supplier	0.500	Retail product
Haematococcus pluvialis	Microalgae	Powder	ALGANEX	Not commercially priced	Provided for research purposes
Nannochloropsis gaditana	Microalgae	Powder	Online retail supplier	0.559	Retail product
Nannochloropsis salina	Microalgae	Powder	Online retail supplier	0.490	Retail product
Porphyridium purpureum	Microalgae	Powder	ALGANEX	Not commercially priced	Provided for research purposes
Schizochytrium limacinum	Microalgae	Powder	Bulk supplier (Alibaba)	0.055	Bulk biomass
Tetraselmis chuii	Microalgae	Powder	ALGANEX	Not commercially priced	Provided for research purposes
Gigartina skottsbergii	Macroalgae (red)	Dried	Online retail supplier	0.666	Retail product

Online retail supplier, Amazon US and eBay US

Supplementary Table S3. Heavy metal and essential mineral composition of selected microalgae and seaweed species and associated safety screening metrics.

a, Concentrations of lead (Pb), total arsenic (As), and total mercury (Hg) reported as mean ± s.d. (μg g^{-1} dry weight), quantified by ICP-OES. N.A. indicates concentrations below instrument detection limits. **b**, Health-based intake benchmarks used for conservative safety screening. **c**, Estimated maximum safe intake (g dry weight per day) calculated using upper-bound concentrations and health-based intake benchmarks for children (see Methods). **d**, Essential mineral concentrations reported as mean ± s.d. (μg g^{-1} dry weight), provided for nutritional context and not used as exclusion criteria.

a. Heavy metals used for safety screening

All concentrations reported as mean ± s.d. (μg g^{-1} dry weight). Total elemental concentrations were quantified by ICP-OES.

Species	Total As	Total Hg	Pb
Arthrospira platensis	N.A.	N.A.	N.A.
Chlorella sp.	N.A.	N.A.	N.A.
Dunaliella salina (A)	N.A.	N.A.	2.48 ± 0.13
Dunaliella salina (B)	N.A.	N.A.	N.A.
Haematococcus pluvialis	N.A.	N.A.	N.A.
Nannochloropsis gaditana	N.A.	N.A.	N.A.
Nannochloropsis salina	N.A.	N.A.	3.36 ± 0.35
Porphyridium purpureum	N.A.	N.A.	N.A.
Schizochytrium limacinum	N.A.	N.A.	N.A.
Tetraselmis chuii	N.A.	N.A.	N.A.
Gigartina skottsbergii	16.03 ± 0.39	N.A.	N.A.

b. Reference intake benchmarks used for screening

Metal	Reference value	Exposure basis	Authority
Lead (Pb)	2.2 μg day^{-1}	Children	US FDA (Interim Reference Level)
Arsenic (inorganic As)	0.3 μg kg^{-1} body weight day^{-1}	Chronic oral exposure	US EPA (RfD)
Mercury (methylmercury)	1.3 μg kg^{-1} body weight week^{-1}	Chronic exposure	EFSA (TWI)

c. Maximum safe intake estimates based on heavy metal screening

Maximum safe intake calculated using upper-bound concentrations (mean + 2 × s.d. for detected values; instrument detection limits for non-detects), assuming a conservative child body weight of 20 kg.

Species	Limiting metal	Maximum safe intake (g DW day^{-1})
Arthrospira platensis	As (below IDL)	≥ 0.71
Chlorella sp.	As (below IDL)	≥ 0.71
Dunaliella salina (A)	Pb	0.8
Dunaliella salina (B)	As (below IDL)	≥ 0.71
Haematococcus pluvialis	As (below IDL)	≥ 0.71
Nannochloropsis gaditana	As (below IDL)	≥ 0.71
Nannochloropsis salina	Pb	0.54
Porphyridium purpureum	As (below IDL)	≥ 0.71
Schizochytrium limacinum	As (below IDL)	≥ 0.71
Tetraselmis chuii	As (below IDL)	≥ 0.71
Gigartina skottsbergii	As	0.36

Essential minerals (reported for nutritional context only)

Values reported as mean ± s.d. (μg g^{-1} DW).

Species	Fe	Zn	Mn
Arthrospira platensis	371.20 ± 0.09	38.71 ± 7.85	15.46 ± 0.03
Chlorella sp.	1212.30 ± 11.67	18.44 ± 6.45	24.31 ± 1.42
Dunaliella salina (A)	1504.33 ± 39.90	193.44 ± 72.25	61.58 ± 2.78
Dunaliella salina (B)	2.78 ± 1.85	14.76 ± 4.57	0.03 ± 0.04
Haematococcus pluvialis	234.70 ± 9.12	13.97 ± 6.23	30.26 ± 1.38
Nannochloropsis gaditana	72.25 ± 4.63	27.44 ± 3.82	34.09 ± 1.26
Nannochloropsis salina	125.58 ± 0.05	114.20 ± 27.61	37.67 ± 0.30
Porphyridium purpureum	1859.33 ± 132.73	69.66 ± 12.84	60.39 ± 4.55
Schizochytrium limacinum	362.12 ± 49.26	125.73 ± 22.66	617.44 ± 85.49
Tetraselmis chuii	2937.42 ± 254.31	62.67 ± 6.93	45.49 ± 3.92
Gigartina skottsbergii	234.06 ± 42.81	20.72 ± 2.84	14.91 ± 4.60

N.A. indicates concentrations below the instrument detection limit (IDL).

Arsenic and mercury concentrations represent total elemental concentrations; speciation of inorganic arsenic or methylmercury was not performed.

Instrument detection limits (ppb in solution) were converted to biomass-based limits assuming a final digest volume of 50 mL and an initial dry biomass mass of 0.1 g.

Maximum safe intake estimates represent conservative screening values and do not constitute dietary recommendations.

Supplementary Table S4. Full compositional profile of selected microalgae and seaweed species used for dietary modeling.

Macronutrients, pigments, and polyphenols were measured experimentally and expressed on a dry weight basis. ALA, EPA, and DHA concentrations were calculated from fatty acid composition and total lipid content and expressed as mg g^{-1} dry weight. Trace element concentrations were used to calculate conservative maximum safe intake values, which were applied as intake constraints in the dietary modeling framework. Maximum safe intake values represent screening thresholds and do not constitute dietary recommendations.

	Protein (% DW)	Carbohydrate (% DW)	Lipid (% DW)	ALA (mg g^{-1} DW)	EPA (mg g^{-1} DW)	DHA (mg g^{-1} DW)	Astaxanthin (mg g^{-1} DW)	beta_carotenoid (mg g^{-1} DW)	VitaminA_RAE (ug g^{-1} DW)	Chlorophyll_A (mg g^{-1} DW)	chlorophyll_B (mg g^{-1} DW)	polyphenol (mg GAE g^{-1} DW)	Fe (mg g^{-1} DW)	Zn (ug g^{-1} DW)	Mn (ug g^{-1} DW)	Si (ug g^{-1} DW)	Limiting metal	Maximum safe intake (g day^{-1})
Arthrospira platensis	35.54	18.14	46.32	0.00	0.00	0.00	0.00	0.00	0.15	2.61	0.01	4.33	371.20	38.71	15.46	0.09	As (below IDL)	NA
Chlorella sp.	36.88	18.60	44.51	206.79	0.00	0.00	ND	ND	ND	2.82	0.43	4.48	1212.30	18.44	24.31	0.31	As (below IDL)	NA
Dunaliella Salina (A)	33.33	28.46	38.21	24.26	0.00	0.00	ND	0.00	0.01	ND	ND	4.24	1504.33	193.44	61.58	0.06	Pb	0.8
Dunaliella Salina (B)	8.68	37.04	54.28	40.71	0.00	0.00	ND	ND	ND	ND	ND	2.98	2.78	14.76	0.03	0.06	As (below IDL)	NA
Haematococcus pluvialis	27.34	23.54	49.12	88.27	0.00	0.00	0.00	0.00	0.01	1.22	0.29	3.82	234.70	13.97	30.26	0.03	As (below IDL)	NA
Nannochloropsis gaditana	32.01	13.06	54.93	0.00	121.29	0.00	0.00	0.00	0.04	3.47	0.88	5.31	72.25	27.44	34.09	0.01	As (below IDL)	NA
Nannochloropsis salina	32.26	15.24	52.50	0.00	178.13	0.00	0.00	0.00	0.01	2.15	0.07	5.81	125.58	114.20	37.67	0.09	Pb	0.54
Porphyridium purpureum	23.39	26.57	50.04	0.00	56.85	0.00	ND	ND	ND	ND	ND	2.51	1859.33	69.66	60.39	0.15	As (below IDL)	NA
Schizochytrium limacinum	21.16	23.23	55.61	0.00	0.00	107.72	ND	ND	ND	ND	ND	11.21	362.12	125.73	617.44	1.21	As (below IDL)	NA
Tetraselmis chuii	35.03	18.84	46.13	105.18	23.90	0.00	ND	ND	ND	3.33	2.14	4.75	2937.42	62.67	45.49	0.07	As (below IDL)	NA
Gigartina skottsbergii	23.72	33.24	43.04	0.00	0.00	0.00	ND	ND	ND	ND	ND	1.80	234.06	20.72	14.91	0.16	As	0.36

ND = not detected; IDL, instrument detection limit

Values reported as % DW are equivalent to g per 100 g dry biomass. Fatty acids, pigments, polyphenols, and minerals are reported on a dry-weight basis.

Supplementary Table S5. Number of children reporting consumption of seaweed or seaweed-containing food items across all CHL survey jurisdictions in 2013, 2015 and 2019.

	Jurisdiction																							
	Palau		Yap		Guam		CNMI		Chuuk		Pohnpei		Kosrae		RMI		Am Samoa		Hawaii		Alaska		Total	
Total Number of Children with Diet Records	n=166		n=190		n=1263		n=1152		n=123		n=187		n=129		n=191		n=1109		n=881		n=664		n=6055	
Food Description	n	%	n	%	n	%	n	%	n	%	n	%	n	%	n	%	n	%	n	%	n	%	n	%
Total (All Seaweed Foods)	15	9.0	1	0.5	45	3.6	80	6.9	0	0.0	0	0.0	7	5.4	0	0.0	32	2.9	200	22.7	22	3.3	402	6.6
Chamorro Sushi (Guamanian)	0	0.0	1	0.5	9	0.7	4	0.3	0	0.0	0	0.0	3	2.3	0	0.0	0	0.0	0	0.0	0	0.0	17	0.3
Musubi [Rice Ball W/ Ume Nori]	2	1.2	0	0.0	0	0.0	0	0.0	0	0.0	0	0.0	0	0.0	0	0.0	0	0.0	9	1.0	0	0.0	11	0.2
Musubi [Rice Ball W/ Ume & Nori]	0	0.0	0	0.0	0	0.0	0	0.0	0	0.0	0	0.0	0	0.0	0	0.0	0	0.0	3	0.3	0	0.0	3	0.0
Musubi, Spam	6	3.6	0	0.0	2	0.2	4	0.3	0	0.0	0	0.0	1	0.8	0	0.0	4	0.4	97	11.0	0	0.0	114	1.9
Seaweed, Furikake	1	0.6	0	0.0	1	0.1	0	0.0	0	0.0	0	0.0	0	0.0	0	0.0	0	0.0	5	0.6	0	0.0	7	0.1
Seaweed, Giant Limu, Fresh	0	0.0	0	0.0	0	0.0	0	0.0	0	0.0	0	0.0	0	0.0	0	0.0	2	0.2	0	0.0	0	0.0	2	0.0
Seaweed, Hijiki, Rehydrated	0	0.0	0	0.0	0	0.0	1	0.1	0	0.0	0	0.0	0	0.0	0	0.0	0	0.0	0	0.0	0	0.0	1	0.0
Seaweed, Konbu, Dried	0	0.0	0	0.0	0	0.0	0	0.0	0	0.0	0	0.0	0	0.0	0	0.0	0	0.0	0	0.0	1	0.2	1	0.0
Seaweed, Konbu, Rehydrated	0	0.0	0	0.0	0	0.0	1	0.1	0	0.0	0	0.0	0	0.0	0	0.0	0	0.0	1	0.1	0	0.0	2	0.0
Seaweed, Nori, Green, Dry	0	0.0	0	0.0	24	1.9	29	2.5	0	0.0	0	0.0	1	0.8	0	0.0	3	0.3	55	6.2	8	1.2	120	2.0
Seaweed, Ogo Nori, Fresh	0	0.0	0	0.0	0	0.0	0	0.0	0	0.0	0	0.0	0	0.0	0	0.0	3	0.3	0	0.0	0	0.0	3	0.0
Seaweed, Tsukudani Nori or Konbu [Seasoned Seaweed]	0	0.0	0	0.0	1	0.1	0	0.0	0	0.0	0	0.0	0	0.0	0	0.0	0	0.0	1	0.1	0	0.0	2	0.0
Seaweed, Waiwaiole, Fresh	0	0.0	0	0.0	0	0.0	1	0.1	0	0.0	0	0.0	0	0.0	0	0.0	1	0.1	0	0.0	0	0.0	2	0.0
Seaweed, Wakame, Rehydrated	0	0.0	0	0.0	0	0.0	0	0.0	0	0.0	0	0.0	0	0.0	0	0.0	0	0.0	2	0.2	1	0.2	3	0.0
Soup, Miso	2	1.2	0	0.0	3	0.2	12	1.0	0	0.0	0	0.0	0	0.0	0	0.0	0	0.0	12	1.4	6	0.9	35	0.6
Soup, Seaweed	0	0.0	0	0.0	0	0.0	2	0.2	0	0.0	0	0.0	0	0.0	0	0.0	0	0.0	0	0.0	0	0.0	2	0.0
Sushi, California Roll	1	0.6	0	0.0	0	0.0	1	0.1	0	0.0	0	0.0	1	0.8	0	0.0	10	0.9	4	0.5	4	0.6	21	0.3
Sushi, Cone	0	0.0	0	0.0	0	0.0	1	0.1	0	0.0	0	0.0	0	0.0	0	0.0	0	0.0	3	0.3	0	0.0	4	0.1
Sushi, Inari	1	0.6	0	0.0	0	0.0	2	0.2	0	0.0	0	0.0	1	0.8	0	0.0	0	0.0	1	0.1	0	0.0	5	0.1
Sushi, Maki	2	1.2	0	0.0	3	0.2	5	0.4	0	0.0	0	0.0	0	0.0	0	0.0	7	0.6	2	0.2	1	0.2	20	0.3
Sushi, Nigiri	0	0.0	0	0.0	2	0.2	10	0.9	0	0.0	0	0.0	0	0.0	0	0.0	1	0.1	4	0.5	1	0.2	18	0.3
Sushi, Teppo or Tekka	0	0.0	0	0.0	0	0.0	7	0.6	0	0.0	0	0.0	0	0.0	0	0.0	1	0.1	1	0.1	0	0.0	9	0.1

Supplementary Table S6. Number of children reporting consumption of seaweed or seaweed-containing food items across Hawaiʻi communities in 2013, 2015 and 2019.

	Hawaii Community													
	Nanakuli		Waimanalo		Hilo		Wailuku		Kauai		Molokai		All HI Communities	
Total Sample of Children with Diet Records	n=232		n=204		n=230		n=198		n=9		n=8		n=881	
Food Description	n	%	n	%	n	%	n	%	n	%	n	%	n	%
Total (All Seaweed Foods)	45	19.4	44	21.6	66	20.6	36	18.2	5	55.6	4	50.0	200	22.7
Musubi [Rice Ball W/ Ume Nori]	5	2.2	3	1.5	1	0.3	0	0.0	0	0.0	0	0.0	9	1.0
Musubi [Rice Ball W/ Ume & Nori]	0	0.0	1	0.5	0	0.0	0	0.0	0	0.0	2	25.0	3	0.3
Musubi, Spam	29	12.5	20	9.8	31	9.7	16	8.1	0	0.0	1	12.5	97	11.0
Seaweed, Furikake	0	0.0	2	1.0	2	0.6	1	0.5	0	0.0	0	0.0	5	0.6
Seaweed, Konbu, Rehydrated	0	0.0	0	0.0	1	0.3	0	0.0	0	0.0	0	0.0	1	0.1
Seaweed, Nori, Green, Dry	9	3.9	11	5.4	17	5.3	15	7.6	2	22.2	1	12.5	55	6.2
Seaweed, Tsukudani Nori or Konbu [Seasoned Seaweed]	0	0.0	0	0.0	0	0.0	1	0.5	0	0.0	0	0.0	1	0.1
Seaweed, Wakame, Rehydrated	0	0.0	0	0.0	1	0.3	1	0.5	0	0.0	0	0.0	2	0.2
Soup, Miso	0	0.0	2	1.0	8	2.5	1	0.5	1	11.1	0	0.0	12	1.4
Sushi, California Roll	0	0.0	2	1.0	1	0.3	1	0.5	0	0.0	0	0.0	4	0.5
Sushi, Cone	0	0.0	2	1.0	1	0.3	0	0.0	0	0.0	0	0.0	3	0.3
Sushi, Inari	1	0.4	0	0.0		0.0	0	0.0	0	0.0	0	0.0	1	0.1
Sushi, Maki	1	0.4	0	0.0	1	0.3	0	0.0	0	0.0	0	0.0	2	0.2
Sushi, Nigiri	0	0.0	1	0.5	1	0.3	0	0.0	2	22.2	0	0.0	4	0.5
Sushi, Teppo or Tekka	0	0.0	0	0.0	1	0.3	0	0.0	0	0.0	0	0.0	1	0.1

Supplementary Table S7. Sensitivity-analysis scenarios used to evaluate the robustness of the constraint-based dietary modeling framework.

Scenario	Biomass cap	Composition input	Trace element constraint
Base case	2.0 g/day	Mean	Conservative screening cap
Low biomass cap	1.0 g/day	Mean	Conservative screening cap
Moderate biomass cap	2.5 g/day	Mean	Conservative screening cap
Lower composition	Mean - SD	Conservative screening cap	Lower-bound nutrient delivery
Upper composition	Mean + SD	Conservative screening cap	Upper-bound nutrient delivery
Relaxed screening cap	Mean	Less restrictive cap	Contaminant assumption sensitivity
